# Synergistic improvement of cinnamylamine production by metabolic regulation

**DOI:** 10.1186/s13036-023-00334-y

**Published:** 2023-02-23

**Authors:** Shan Yuan, Chao Xu, Miaomiao Jin, Mo Xian, Wei Liu

**Affiliations:** 1 CAS Key Laboratory of Biobased Materials, Qingdao Institute of Bioenergy and Bioprocess Technology, Chinese Academy of Sciences, No. 189 Songling Road, Qingdao, 266101 Shandong P.R. China; 2 University of Chinese Academy of Sciences, Beijing, P.R. China

**Keywords:** Cinnamylamine, Metabolic engineering, Transcriptome analysis, *Escherichia coli*, One-pot, Whole-cell catalysis

## Abstract

**Background:**

Aromatic primary amines (APAs) are key intermediates in the chemical industry with numerous applications. Efficient and mild biocatalytic synthesis is an excellent complement to traditional chemical synthesis. Our lab previously reported a whole-cell catalytic system for the synthesis of APAs catalyzed by carboxylic acid reductase from *Neurospora crassa* (ncCAR) and ω-transaminase from *Ochrobactrum anthropi* (OATA). However, the accumulation of toxic intermediates (aromatic aldehydes) during biocatalytic synthesis affected yields of APAs due to metabolic imbalance.

**Results:**

In this work, the biocatalytic synthesis of APAs (taking cinnamylamine as an example) was metabolically regulated by the overexpression or knockout of five native global transcription factors (TFs), the overexpression of eight native resistance genes, and optimization of promoters. Transcriptome analysis showed that knockout of the TF *arcA* increased the fluxes of NADPH and ATP in *E. coli*, while the rate of pyruvate metabolism was accelerated. In addition, the genes related to stress and detoxification were upregulated with the overexpression of resistance gene *marA*, which reduced the NADPH level in *E. coli*. Then, the expression level of soluble OATA increased by promoter optimization. Overall, *arcA* and *marA* could regulate the catalytic rate of NADPH- dependent ncCAR, while *arcA* and optimized promoter could regulate the catalytic rate of OATA. Lastly, the cinnamylamine yield of the best metabolically engineered strain S020 was increased to 90% (9 mM, 1.2 g/L), and the accumulation of cinnamaldehyde was below 0.9 mM. This work reported the highest production of cinnamylamine by biocatalytic synthesis.

**Conclusion:**

This regulatory process provides a common strategy for regulating the biocatalytic synthesis of other APAs. Being entirely biocatalytic, our one-pot procedure provides considerable advantages in terms of environmental and safety impacts over reported chemical methods.

**Supplementary Information:**

The online version contains supplementary material available at 10.1186/s13036-023-00334-y.

## Introduction

**Fig. 1:**
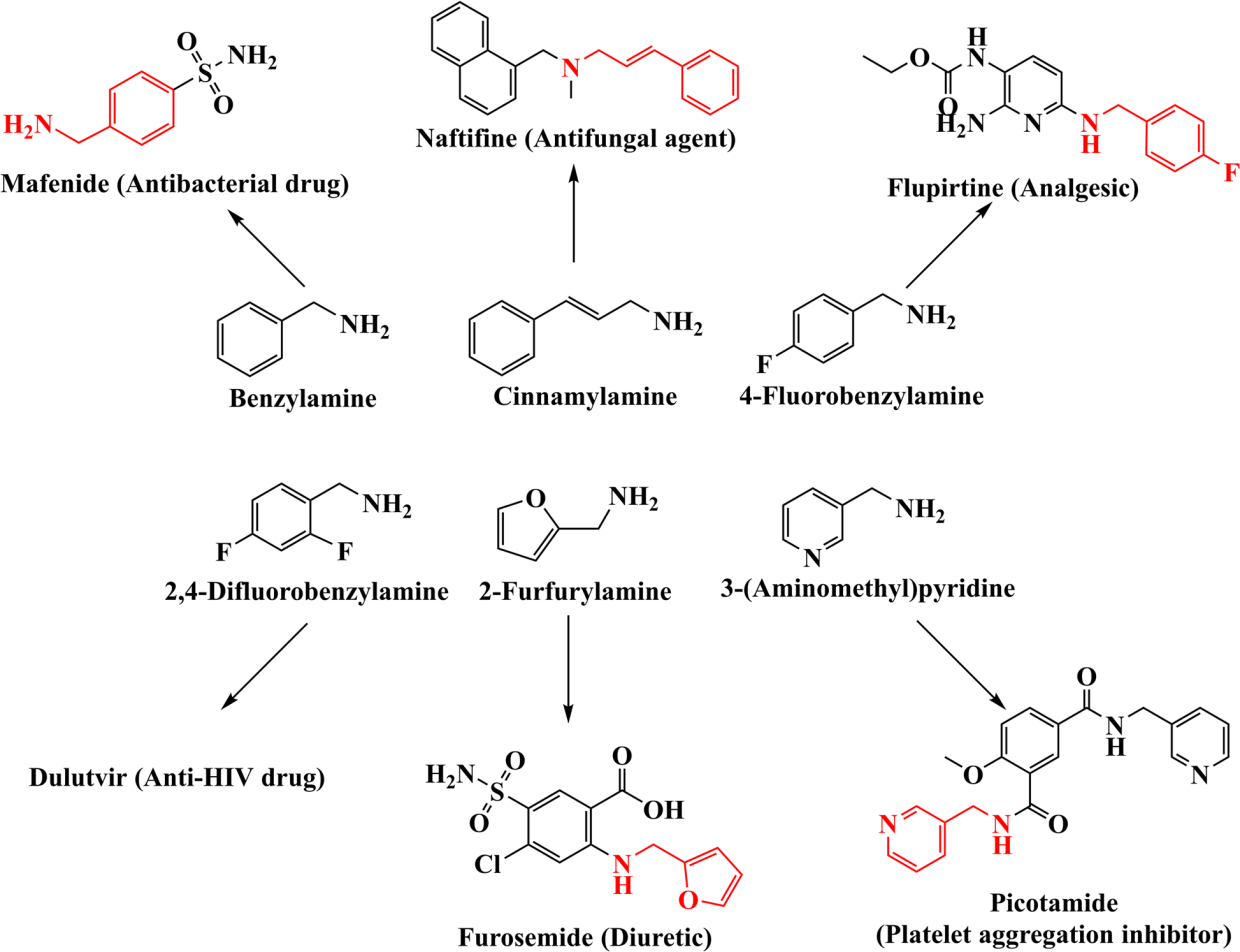
Applications of several APAs

APAs are key intermediates in the chemical industry with extensive applications in the manufacture of pharmaceuticals, pesticides, polymers, dyes and detergents (Fig. 1) [1–5]. For example, cinnamylamine can be used to synthesize the antifungal agent naftifine for the treatment of infections of Tinea, Trichophyton, and Epidermophyton species [6]. 4-Fluorobenzylamine is an intermediate of the analgesic flupirtine [7]. 2-Furfurylamine can be used to make the powerful diuretic furosemide [8]. 2,4-Difluorobenzylamine is a key intermediate of dulutvir, which is a new anti-HIV drug under GlaxoSmithKline (GSK) [9].

At present, the industrial synthesis method of APAs is mainly chemical synthesis [1, 2]. The industrial route of APAs mainly uses corresponding nitriles, aldehydes and alcohols as raw materials to synthesize APAs. For example, cinnamylamine and 3-(aminomethyl)pyridine (3-AMP) are synthesized from corresponding nitriles [10, 11], and 2-furfurylamine is synthesized from 2-furfural (Fig. S1) [12]. These processes are characterized by the use of metal catalysts (Co, Cu, etc.), organic solvents (methanol, etc.), or harsh energy-demanding conditions (high temperature or pressure) with expensive safety measures.

**Scheme 1:**
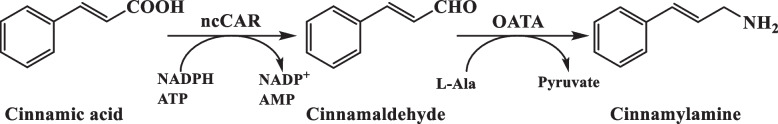
The one-pot biocatalytic route for the synthesis of APAs (taking cinnamylamine as an example)

Biocatalytic synthesis takes advantage of multistep enzymatic reactions that efficiently convert cheap and renewable substrates into value-added chemicals under mild reaction conditions [13–16], and it is emerging as an economic and environmentally friendly supplement to traditional chemical approaches. There are few reports on biocatalytic synthesis of APAs. Our lab previously reported a whole-cell catalytic route for the synthesis of APAs (such as cinnamylamine) from carboxylic acids (Scheme 1) [17]. APAs was synthesized for the first time by using biocatalysts co-expressing carboxylic acid reductase from *Neurospora crassa* (ncCAR), ω-transaminase from *Ochrobactrum anthropi* (OATA) and phosphopantetheine transferase from *E. coli* (PPTase, modified ncCAR into holoenzyme [18]) in one pot [17]. Some substrates in this route, such as benzoic acid and cinnamic acid, can be synthesized de novo [19, 20]. Therefore, this route had the benefits of low cost and environmentally friendly. However, there are some disadvantages in this route. On the one hand, the metabolic imbalance of the two enzymes in this process could easily lead to the accumulation of toxic intermediates (aromatic aldehydes), which was not conducive to the continuous production of products. On the other hand, the yields of some APAs were still low, even with prolonged reaction times.

Metabolic balance of strains often refers to the balance of reactions catalyzed by two enzymes. The goal of this metabolic balance is that no intermediates are produced. The metabolic balance can be regulated by metabolic engineering of strains to increase the yield and reduce the accumulated intermediates [21, 22]. There are currently few studies on the metabolic regulation of the biocatalytic synthesis of APAs. In this study, the biocatalytic synthesis of APAs (taking cinnamylamine as an example) was metabolically regulated to improve the yield of APAs. After metabolic engineering and optimization of reaction conditions, the yield of cinnamylamine and conversion of cinnamic acid reached 90 and 100%, respectively, which was comparable to chemical methods. Furthermore, as demonstrated by transcriptome analysis and proteomic approach, the increased yield and conversion was due to the increased supply of NADPH and ATP and the balance of expression levels of the two enzymes. This metabolic regulatory process is theoretically suitable for regulating the biocatalytic synthesis of other APAs.

## Materials and methods

### Materials

Cinnamic acid and other chemicals were obtained from Aladdin (Shanghai, China). A DNA gel extraction kit, plasmid purification kit, Primer STAR Max and DNA marker were obtained from TAKARA (Japan). Protein markers, restriction endonucleases and T4 DNA ligase were obtained from Thermo Fisher Scientific (USA). M9 Minimal Salts (M9 buffer) were obtained from Sangon Biotech (Shanghai, China). *E. coli* RARE(DE3) was purchased from Addgene (USA).

### Construction of plasmids and strains

The plasmids and strains used in this study are listed in Table 1.

**Table 1:** Plasmids or strains used in this study

No.	Plasmids or strains	Description	Source
p001	pET28a-*nccar-pptase*	T7, *nccar* with His tag, T7, *pptase*, lacIq, pBR322 ori, Kanr	Laboratory stock
p002	pACYCDuet1-*oata*	T7, *oata*, lacIq, pBR322 ori, Cmr	Laboratory stock
p003	pACYCDuet1-*oata-arcA*	T7, *oata*, T7, *arcA*, lacIq, pBR322 ori, Cmr	This study
p004	pACYCDuet1-*oata-cra*	T7, *oata*, T7, *cra*, lacIq, pBR322 ori, Cmr	This study
p005	pACYCDuet1-*oata-crp*	T7, *oata*, T7, *crp*, lacIq, pBR322 ori, Cmr	This study
p006	pACYCDuet1-*oata-fnr*	T7, *oata*, T7, *fnr*, lacIq, pBR322 ori, Cmr	This study
p007	pACYCDuet1-*oata-csrA*	T7, *oata*, T7, *csrA*, lacIq, pBR322 ori, Cmr	This study
p008	pACYCDuet1-*oata-marA*	T7, *oata*, T7, *marA*, lacIq, pBR322 ori, Cmr	This study
p009	pACYCDuet1-*oata-rob*	T7, *oata*, T7, *rob*, lacIq, pBR322 ori, Cmr	This study
p010	pACYCDuet1-*oata-soxS*	T7, *oata*, T7, *soxS*, lacIq, pBR322 ori, Cmr	This study
p011	pACYCDuet1-*oata-csgD*	T7, *oata*, T7, *csgD*, lacIq, pBR322 ori, Cmr	This study
p012	pACYCDuet1-*oata-emrE*	T7, *oata*, T7, *emrE*, lacIq, pBR322 ori, Cmr	This study
p013	pACYCDuet1-*oata-emrB*	T7, *oata*, T7, *emrB*, lacIq, pBR322 ori, Cmr	This study
p014	pACYCDuet1-*oata-rpoS*	T7, *oata*, T7, *rpoS*, lacIq, pBR322 ori, Cmr	This study
p015	pACYCDuet1-*oata-tolC*	T7, *oata*, T7, *tolC*, lacIq, pBR322 ori, Cmr	This study
p016	pACYCDuet1-P_1,6_-*oata*	P_1,6_, *oata*, lacIq, pBR322 ori, Cmr	This study
p017	pACYCDuet1-P_2,51_-*oata*	P_2,51_, *oata*, lacIq, pBR322 ori, Cmr	This study
p018	pACYCDuet1-P_1,6_-*oata*-*marA*	P_1,6_, *oata*, T7, *marA*, lacIq, pBR322 ori, Cmr	This study
S001	*E. coli* RARE(DE3)	MG1655(DE3) *∆dkgB ∆yeaE ∆(yqhC-dkgA) ∆yahK ∆yjgB ∆yqhD*	Addgene
S002	*E. coli* RARE(DE3) *∆arcA*	MG1655(DE3) *∆dkgB ∆yeaE ∆(yqhC-dkgA) ∆yahK ∆yjgB ∆yqhD ∆arcA*	Laboratory stock
S003	RARE(DE3)/ pET28a-*nccar-pptase*/pACYCDuet1-*oata*	RARE(DE3) carrying p001 and p002	This study
S004	RARE(DE3)/ pET28a-*nccar-pptase*/pACYCDuet1-*oata-arcA*	RARE(DE3) carrying p001 and p003	This study
S005	RARE(DE3)/ pET28a-*nccar-pptase*/pACYCDuet1-*oata-cra*	RARE(DE3) carrying p001 and p004	This study
S006	RARE(DE3)/ pET28a-*nccar-pptase*/pACYCDuet1-*oata-crp*	RARE(DE3) carrying p001 and p005	This study
S007	RARE(DE3)/ pET28a-*nccar-pptase*/pACYCDuet1-*oata-fnr*	RARE(DE3) carrying p001 and p006	This study
S008	RARE(DE3)/ pET28a-*nccar-pptase*/pACYCDuet1-*oata-csrA*	RARE(DE3) carrying p001 and p007	This study
S009	RARE(DE3)△*arcA* / pET28a-*nccar-pptase*/pACYCDuet1-*oata*	RARE(DE3)△*arcA* carrying p001 and p002	This study
S010	RARE(DE3)/ pET28a-*nccar-pptase*/pACYCDuet1-*oata-marA*	RARE(DE3) carrying p001 and p008	This study
S011	RARE(DE3)/ pET28a-*nccar-pptase*/pACYCDuet1-*oata-rob*	RARE(DE3) carrying p001 and p009	This study
S012	RARE(DE3)/ pET28a-*nccar-pptase*/pACYCDuet1-*oata-soxS*	RARE(DE3) carrying p001 and p010	This study
S013	RARE(DE3)/ pET28a-*nccar-pptase*/pACYCDuet1-*oata-csgD*	RARE(DE3) carrying p001 and p011	This study
S014	RARE(DE3)/ pET28a-*nccar-pptase*/pACYCDuet1-*oata-emrE*	RARE(DE3) carrying p001 and p012	This study
S015	RARE(DE3)/ pET28a-*nccar-pptase*/pACYCDuet1-*oata-emrB*	RARE(DE3) carrying p001 and p013	This study
S016	RARE(DE3)/ pET28a-*nccar-pptase*/pACYCDuet1-*oata-rpoS*	RARE(DE3) carrying p001 and p014	This study
S017	RARE(DE3)/ pET28a-*nccar-pptase*/pACYCDuet1-*oata-tolC*	RARE(DE3) carrying p001 and p015	This study
S018	RARE(DE3)/ pET28a-*nccar-pptase*/pACYCDuet1-P_1,6_-*oata*	RARE(DE3) carrying p001 and p016	This study
S019	RARE(DE3)/ pET28a-*nccar-pptase*/pACYCDuet1-P_2,51_-*oata*	RARE(DE3) carrying p001 and p017	This study
S020	RARE(DE3)△*arcA* / pET28a-*nccar-pptase*/pACYCDuet1-P_1,6_-*oata*	RARE(DE3)△*arcA* carrying p001 and p016	This study
S021	RARE(DE3)△*arcA* / pET28a-*nccar-pptase*/pACYCDuet1-*oata-marA*	RARE(DE3)△*arcA* carrying p001 and p008	This study
S022	RARE(DE3)△*arcA* / pET28a-*nccar-pptase*/pACYCDuet1-P_1,6_-*oata-marA*	RARE(DE3)△*arcA* carrying p001 and p018	This study

The heterologous genes used in this study have been codon-optimized prior to in vivo expression in *E. coli*. The recombinant plasmids, pET28a-*nccar-pptase* and pACYCDuet1-*oata*, were constructed in previous report [17]. The Gene ID of these regulatory factors and resistance genes used in this study are as follows: *arcA*: 948874; *cra*: 944804; *crp*: 947867; *csrA*: 947176; *fnr*: 945908; *marA*: 947613; *rob*: 948916; *soxS*: 948567; *emrE*: 948442; *emrB*: 947167; *tolC*: 947521; *rpoS*: 947210; *csgD*: 949119. These genes were synthesized by the Beijing Genomics Institute (Beijing, China). The synthesized genes were digested with *Nde*I and *Bgl*II, and the digested fragment was ligated into the pACYCDuet1-*oata*, which was digested with the same restriction enzymes. The P_1, 6_ and P_2, 51_ promoter were synthesized by the Beijing Genomics Institute (Table S1). The T7 promoter and lac operator of *oata* were replaced by synthesized P_1,6_ or P_2,51_ to obtain p016 and p017. The constructed vector was transformed into *E. coli* RARE (DE3), reported by Aditya M. Kunjapur et al., which has knocked out *dkgB* (Gene ID: 944901), *yeaE* (Gene ID: 946302), *dkgA* (Gene ID: 947495), *yqhC* (Gene ID: 947491), *yqhD* (Gene ID: 947493), *yjgB* (Gene ID: 948802) and *yahK* (Gene ID: 944975) [23]. Single knockout strains (Δ*arcA*) were obtained from the Keio collection, which was derived from *E. coli* K-12 BW25113 [24, 25].

### Culture of strains

*E. coli* cells were incubated at 37 °C for 12 h in 5 mL of LB medium containing 50 μg/mL Kan and 34 μg/mL Cm. The grown cells (2 mL) were then transferred into 200 mL of LB medium containing appropriate antibiotics and cultivated at 37 °C in the thermostatic incubator (ZHICHENG ZWYR-D2403, China). When OD_600 nm_ reached 0.6, 0.2 mM IPTG (isopropyl beta-D-1-thiogalactopyranoside) was added. The cells were harvested after 15 h by centrifugation (Himac CR21N, Japan) at 5000 rpm for 5 min. Collected cells were immediately used for whole-cell catalysis, or frozen at − 80 °C for transcriptome analysis.

### Whole-cell bioconversion

The cells were washed with M9 buffer (15.12 g/L Na_2_HPO_4_·12H_2_O, 3 g/L KH_2_PO_4_, 0.5 g/L NaCl and 1 g/L NH_4_Cl, pH 7.0) to remove residual culture media and further resuspended in M9 buffer. The typical assay to directly measure reaction products was performed as follows: wet whole cells (OD_600 nm_ = 30), 10 mM cinnamic acid, 20 mM L-alanine (L-Ala) and 20 mM MgSO_4_. Our previous studies have demonstrated that the by-product (pyruvate) produced during the reaction can act as a carbon source, thus eliminating the need for additional glucose [17]. Reactions were performed in M9 buffer (pH 7.0) at 37 °C with persistent stirring at 200 rpm. After centrifugation, the supernatants were analyzed by HPLC. A 500 mM cinnamic acid solution (dissolved in DMSO) was diluted 100-fold, and then the initial niacin concentration was tested by HPLC. The yield, conversion, selectivity and productivity were calculated. The calculation formulas are as follows: “Yield” = molar concentration of cinnamylamine/molar concentration of input cinnamic acid (expressed as %); “Conversion” = molar concentration of consumed cinnamic acid/molar concentration of input cinnamic acid (expressed as %); “Selectivity” = molar concentration of cinnamylamine/molar concentration of consumed cinnamic acid (expressed as %); “Productivity” = concentration of cinnamylamine (g/L)/ reaction time (h).

### Protein expression and SDS-PAGE analysis

The cells were harvested by centrifugation (Himac CR21N, Japan) at 5000 rpm for 5 min and washed with 20 mM Tris-HCl buffer (pH 7.5). After the cells were disrupted by a high-pressure cell cracker (Constant systems One shot 40KPSI, England), the cell debris was discarded by centrifugation (10,000 rpm, 30 min, 4 °C). Sodium dodecyl sulfate polyacrylamide gel electrophoresis (SDS-PAGE, 12%) was used to assess the protein expression.

### HPLC analysis

HPLC analyses were performed on the HPLC system (ThermoFisher UltiMate 3000, USA). For cinnamylamine, cinnamic acid, cinnamaldehyde and cinnamic alcohol, HPLC conditions were as follows: a ZORABX-C18 column (Agilent, USA) was used with a mobile phase of H_2_O containing 0.1% TFA (phase A) and acetonitrile containing 0.1% TFA (phase B); isocratic elution (1:1) was performed for 20 min at a flow rate of 1 mL/min with a column temperature of 30 °C.

### Transcriptome analysis

Samples were subjected to transcriptome analysis by a testing company. Strains S003 (wild type strain, WT), S009 and S010 were selected for transcriptome sequencing using IlluminaHiSeq platform, and the quality of the raw data (reads) was evaluated. The percentage of Q30 bases in each sample was ≥92.02%, and the GC content was 49.94% ~ 51.46%. This indicates that the sequencing quality and data reliability were high. The |log_2_(foldchange)| ≥1 and *P*-value < 0.05 were assigned as differentially expressed.

Functional annotation of differentially expressed reads was performed by searching the Gene Ontology (GO) Consortium and Kyoto Encyclopedia of Genes and Genomes (KEGG) databases. Enrichment analysis of differentially expressed genes (DEGs) were performed in the GO database. KEGG was used for statistical enrichment of DEGs in KEGG pathways. Specific methods for transcriptome analysis are presented in “Supplementary methods for transcriptome analysis” in “Supplementary Information”.

## Results and discussion

### Effects of global transcription factors on the biocatalytic synthesis of cinnamylamine

**Fig. 2:**
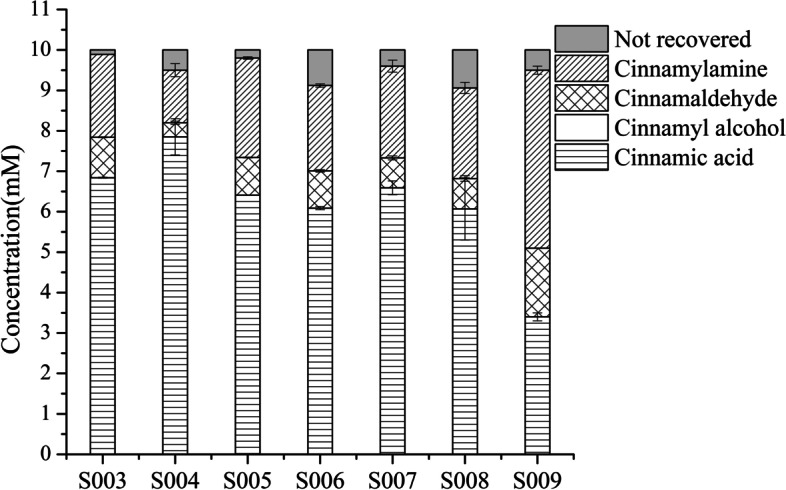
Effects of five global TFs on the conversion of cinnamic acid to cinnamylamine. The reaction was performed in M9 buffer (pH 7.0) containing 10 mM cinnamic acid (the initial concentration); 20 mM L-Ala; 20 mM MgSO_4_; S003-S009 wet cells, OD_600 nm_ = 30 at 30 °C with 200 rpm shaking for 1 h. Not recovered: undetectable cinnamic acid. It may be converted to other unknown byproducts. According to our previous report [17], the by-product pyruvate generated during the reaction can replace glucose as the carbon source for the reaction (Scheme 1)

In this study, five native global transcription factors (TFs) involved in carbon metabolism were overexpressed, including four DNA-binding transcriptional dual regulators *arcA*, *cra*, *crp* and *fnr*, and one carbon storage regulator *csrA*. The five TFs were overexpressed in strain S003 (WT), respectively, to obtain strains S004-S008. The titer of cinnamylamine in strains S003-S008 was detected by HPLC. The results showed that the overexpression of *cra*, *crp*, *csrA* and *fnr* had little effect on the production of cinnamylamine and the accumulation of cinnamaldehyde. However, overexpression of *arcA* significantly reduced the yield of cinnamylamine by 37% (Fig. 2, Table 2). Thus, the *arcA* of S003 was knocked out to obtain S009. The results showed that knocking out *arcA* increased the yield (and productivity) of cinnamylamine, which was 2.1 times that of S003 (Fig. 2, Table 2). Although the accumulation of cinnamaldehyde also increased, the selectivity increased. According to our previous report, knocking out *arcA* increased the yield of 3-AMP, however, the reason was not analyzed experimentally [17].

**Table 2:** Effects of global TFs on the yield, conversion, selectivity and productivity

Strains	Yield%	Conversion%	Selectivity %	Productivity (g/L/h)
S003	20.5	31.6	64.9	0.27 ± 0.000
S004	13	21.5	60.5	0.17 ± 0.021
S005	24.6	35.9	68.5	0.33 ± 0.004
S006	21.1	39.1	54	0.28 ± 0.005
S007	22.7	34.1	66.6	0.3 ± 0.02
S008	22.4	39.3	57	0.3 ± 0.019
S009	44	66	66.7	0.59 ± 0.013

The reaction conditions were the same as in Fig. 2

**Fig. 3:**
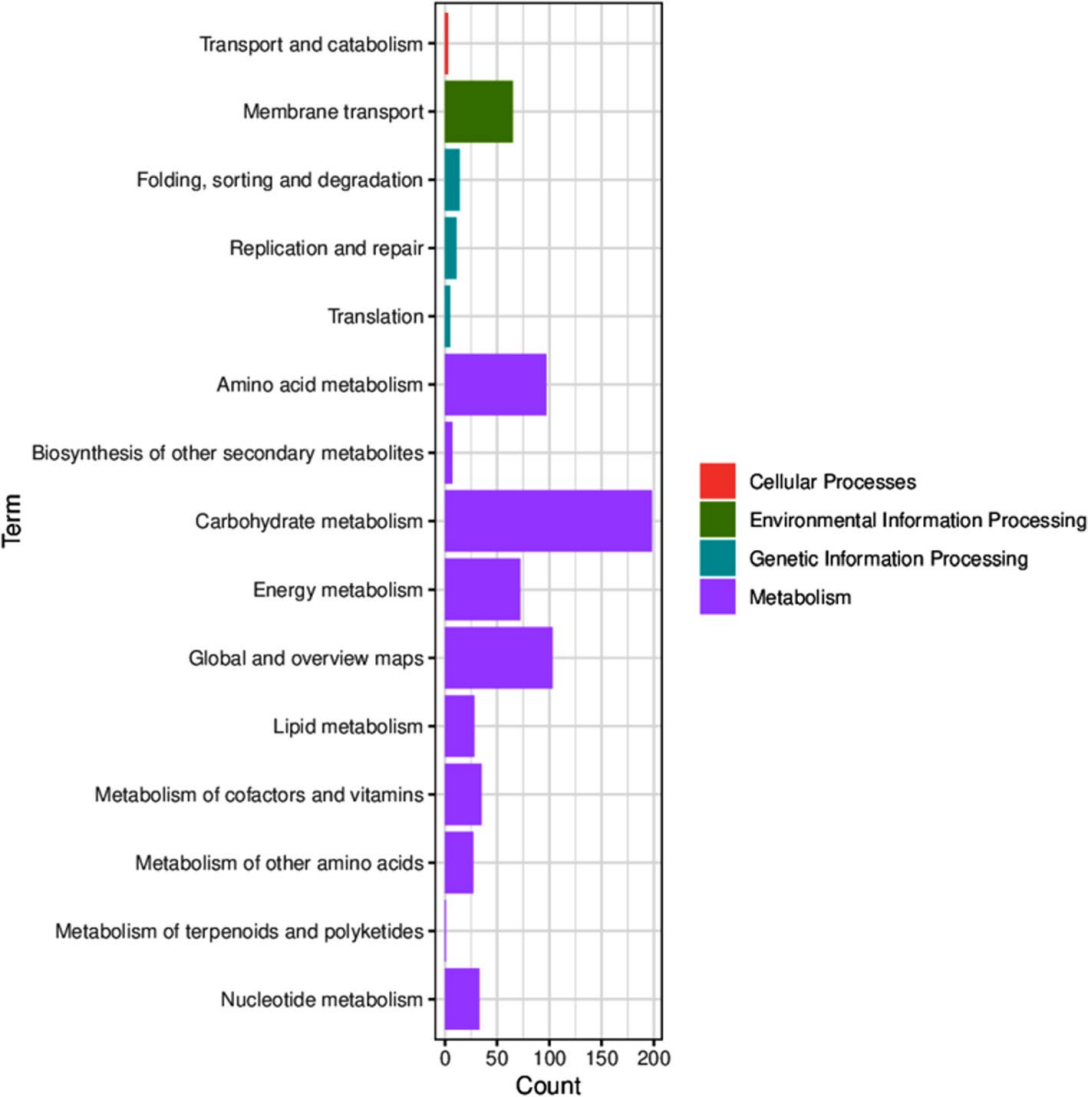
KEGG classification analysis of DEGs between S003 and S009. The ordinate is the name of the KEGG metabolic pathway, and the abscissa is the number of genes annotated to this pathway and the ratio of the number of genes to the total number of genes annotated

The reason that knockout of *arcA* resulted in increased cinnamylamine production was analyzed by transcriptome analysis. ArcA is a global regulator that regulates multiple metabolic pathways [26], so there are many genes that are affected. A total of 1266 genes were differentially expressed (|log_2_ fold change| > 1 and *P*-value < 0.05) between S003 and S009, of which 676 were upregulated (Fig. S2). The KEGG annotation results of the DEGs were classified according to KEGG pathway type, with a total of 6 categories. The classification chart is shown in Fig. 3, which shows that the proportion of carbohydrate metabolism was the highest in the metabolic category. ArcA is one of the major regulators of the *E. coli* respiratory pathway in response to redox conditions, and the main targets of *arcA* are genes involved in central carbon metabolism [26], which is consistent with the results of transcriptome analysis. The only carbon source in the reaction system is the by-product, pyruvate. According to our previous report, pyruvate can replace glucose to provide carbon source for the reaction [17]. Therefore, knocking out *arcA* will regulate metabolic pathways involving pyruvate.

**Fig. 4:**
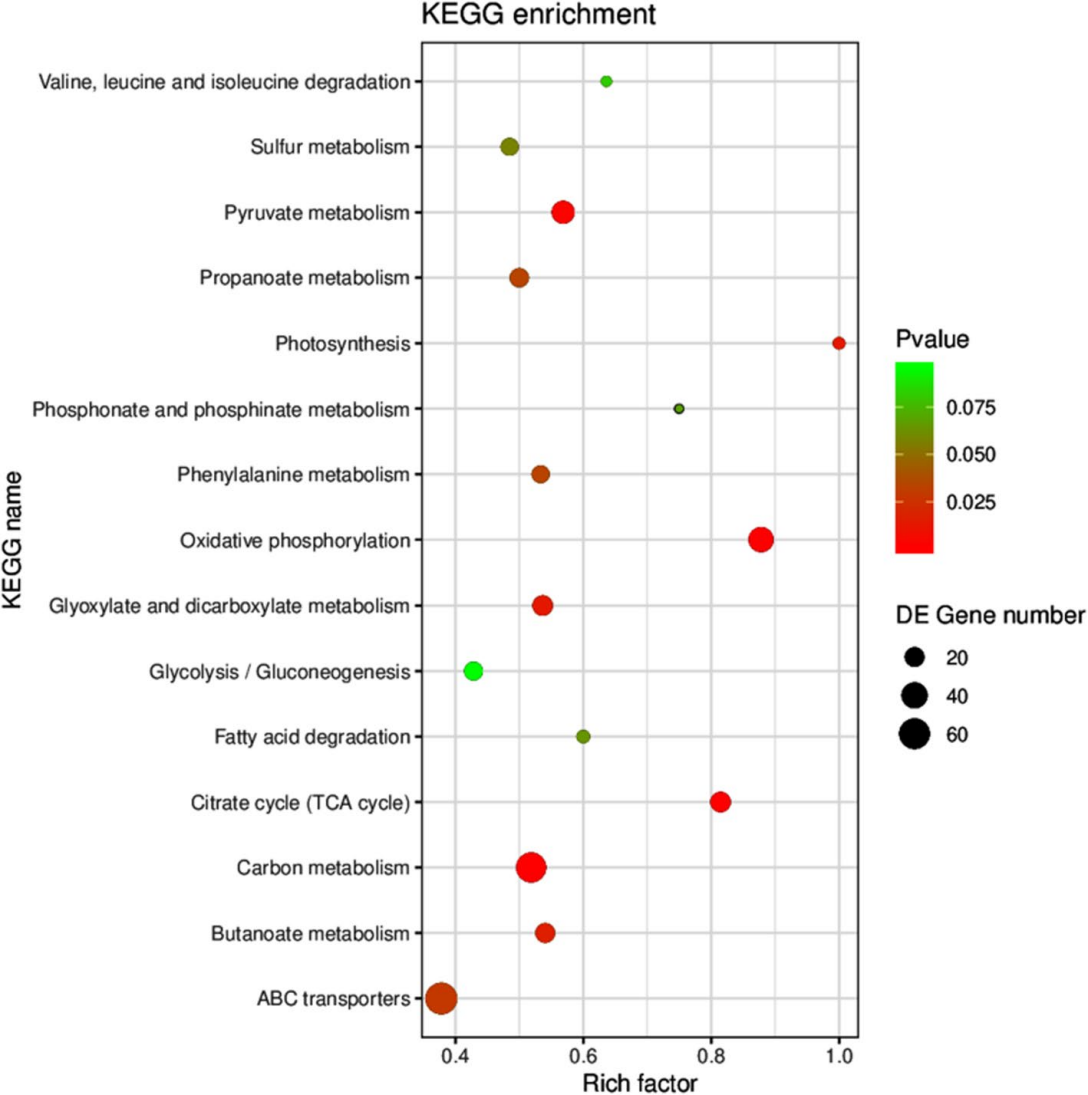
KEGG enrichment analysis of DEGs between S003 and S009. Each circle in the figure represents a KEGG pathway. The ordinate represents the pathway name, and the abscissa represents the enrichment factor. It represents the ratio of the proportions of DEGs annotated to a certain pathway to the total proportion of genes annotated to this pathway. The higher the enrichment factor is, the more significant the enrichment level of DEGs in this pathway. The color of the circle represents the Q value, which is the *P* value after the correction of multiple hypothesis tests. The smaller the Q value is, the more reliable the enrichment significance of DEGs in this pathway. The size of the circle indicates the number of genes enriched in the pathway, and the larger the circle is, the greater the number of genes

To better understand the molecular mechanisms in *arcA*, functional enrichment analysis was performed on the DEGs. The results of enrichment analysis of the KEGG pathways of DEGs are shown in Fig. 4. DEGs are mainly enriched in 3 pathways, including TCA cycle, oxidative phosphorylation and pyruvate metabolism. The vast majority of DEGs of the 3 pathways are significantly upregulated genes (Figs. S3, S4 and S5).

TCA cycle is the main pathway for supplying NAD(P)H and ATP, so the upregulation of this pathway increases the flux of NAD(P)H and ATP in S009. Specifically, genes that generated NAD(P)H and ATP in the TCA cycle (such as pyruvate dehydrogenase gene, NAD(P)-isocitrate dehydrogenase gene, succinate dehydrogenase gene, alpha-ketoglutarate dehydrogenase gene) were upregulated (log_2_ fold change = 1, 2.6, 2.5, 3.5, respectively) in S009 compared to S003 (Fig. S3). The expression level of soluble pyridine nucleotide transhydrogenase gene (*sth*), which catalyzed NADH and NADP^+^ to generate NADPH and NAD^+^, was also increased by 2-fold in S009. The up-regulation of these genes is beneficial to the supply of NAD(P)H and ATP in S009. Previous reports have shown that knockout of the *arcA* activated the transcription levels of the TCA cycle genes and increased the NADH/NAD^+^ ratio and ATP production rate [26, 27]. Therefore, knockout of *arcA* accelerated the supply of NAD(P)H and ATP in line with these reports.

Oxidative phosphorylation is the process of releasing energy when the substance is oxidized and supplying the energy to ADP to synthesize ATP. Therefore, the upregulation of this pathway accelerated the synthesis of ATP in S009. Specifically, the cytochrome bo gene (*cyo*) and NADH dehydrogenase-I gene (*nuo*) used to drive ATP synthesis in this pathway were upregulated (log_2_ fold change = 1.5, 2, respectively) (Fig. S4), which favored ATP synthesis and accelerated ncCAR-catalyzed reaction.

Upregulation for the pyruvate metabolic pathway accelerated the metabolism of the by-product pyruvate (Fig. S5). OATA is a reversible ω-transaminase, and the accelerated metabolism of pyruvate promotes the synthesis of cinnamylamine.

In a word, the upregulation of 3 pathways is also beneficial for the bioconversion of cinnamyl acid to cinnamylamine. Therefore, transcriptome analysis is a good explanation for the reason why *arcA* accelerates the yield of cinnamylamine. Previous reports showed that the knockout of *arcA* increased the production of poly(3-hydroxyalkanoate) [28], phloroglucinol [29], and ethylene glycol [30] by 64-fold, 2-fold, and 54%, respectively. The biosynthesis of these compounds requires consumption of NAD(P)H or ATP. Therefore, knockout of *arcA* may promote the synthesis of compounds that require depletion of NAD(P)H and ATP.

### Effects of resistance genes on the biocatalytic synthesis of cinnamylamine

The intermediate cinnamaldehyde have been proved to be toxic to *E. coli* [19], which inhibits the activity of S009. Therefore, we intend to address this issue by reducing aldehyde accumulation or increasing cellular resistance to aldehydes.

Eight native resistance genes included three local regulators and five multidrug resistance transporters (MDTs). The native local regulators of RARE(DE3), such as *marA*, *rob*, and *soxS*, can regulate the transcription of various antibiotic and superoxide resistance genes to promote the conversion of toxic substances into non-toxic substances. Three genes were overexpressed in strain S003 to obtain S010-S012. In addition, bacterial multidrug resistance transporters (MDTs) are responsible for the extracellular transporting of toxic substrates from inside the cells to increase cellular resistance to these substances [31]. Then, native MDTs *csgD*, *emrE*, *emrB*, *rpoS* and *tolC* were overexpressed in S003 to obtain S013-S017, respectively.

**Fig. 5:**
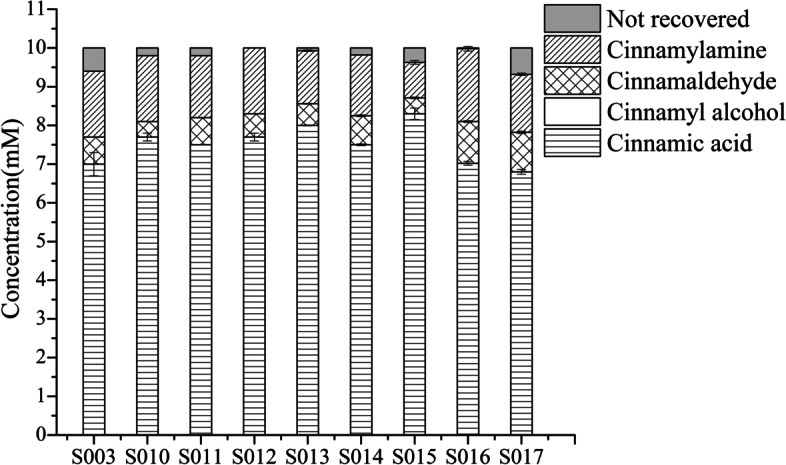
Effects of 8 resistance genes on the conversion of cinnamic acid to cinnamylamine. The reaction was performed in M9 buffer (pH 7.0) containing 10 mM cinnamic acid; 20 mM L-Ala; 20 mM MgSO_4_; S003 or S010-S017 wet cells, OD_600 nm_ = 30 at 30 °C with 200 rpm shaking for 1 h

Although the yield of cinnamylamine did not increase after overexpression of MarA and SoxS, the accumulation of intermediate cinnamaldehyde decreased to 45 and 55% of that of WT, which will reduce the toxicity of cinnamaldehyde to cells (Fig. 5, Table 3). The overexpression of EmrB (S015) resulted in decreased concentrations of cinnamaldehyde and cinnamylamine, which was unfavorable. Effects of overexpression of other genes was not obvious.

**Table 3:** Effects of resistance genes on the yield, conversion, selectivity and productivity

Strains	Yield%	Conversion%	Selectivity %	Productivity (g/L/h)
S003	17	30	56.7	0.23 ± 0.000
S010	17	23	73.9	0.23 ± 0.000
S011	16	25	64	0.21 ± 0.000
S012	17	23	73.9	0.23 ± 0.000
S013	13.7	20	68.5	0.18 ± 0.003
S014	15.7	25	62.8	0.21 ± 0.000
S015	9.2	17	54.1	0.12 ± 0.007
S016	18.8	29.8	63.1	0.25 ± 0.008
S017	15	32	46.9	0.2 ± 0.004

The reaction conditions were the same as in Fig. 5

The reason that overexpression of *marA* resulted in decreased accumulation of cinnamaldehyde was analyzed by transcriptome analysis. MarA, a local regulator, regulates several multidrug resistance genes, so there are not many genes affected. A total of 41 genes were differentially expressed in S003 and S010, of which 33 were upregulated (Fig. S6). DEG analysis showed that overexpression of *marA* resulted in upregulation of genes related to stress, detoxification, and antioxidant effects, such as NAD(P)H nitroreductase gene (*nfnB*/*nfsA*), luciferase-like monooxygenase gene (*yhbW*), NADPH:quinone oxidoreductase gene (*mdaB*), superoxide dismutase gene (Mn) (*sod2*) and aconitate hydratase gene (*acnA*) in S010 (Table 4), compared with S003. The Ecocyc database (https://ecocyc.org/) shows that *marA* can activate the transcription of detoxification or stress genes such as *nfnB*/*nfsA*, which is consistent with the experimental results. Many enzymes(such as nfnB/nfsA, mdaB) involved in oxidative stress responses have been reported to reduce the intracellular NADPH level in *E. coli*, which lowers the reducing power of the cells [32]. Therefore, the NADPH level in S010 cells is reduced compared with S003, which is responsible for the lower conversion of cinnamic acid to cinnamaldehyde. As a result, the accumulation of cinnamaldehyde was reduced. However, because cinnamaldehyde remained (0.4 mM) (Fig. 5), it did not affect the conversion of cinnamaldehyde to cinnamylamine (similar yield and productivity).

**Table 4:** Up-regulated genes related to stress, detoxification, and antioxidant effects in DEGs

Classification	Name	Log_2_ FC^a^	Description/Function
Detoxification	nfnB/nfsA	3.1/2.8	NAD(P)H nitroreductase/ Related to drug activation and detoxification
	yhbW	2.8	Luciferase-like monooxygenase/ Related to the metabolism of drugs and poisons
	mdaB	1.8	NADPH:quinone oxidoreductase/ Improved resistance to cytotoxic drugs
	SOD2	3.9	Superoxide dismutase (Mn)/ Eliminated harmful substances produced by organisms during metabolism
Antioxidation	HycG	3.0	Formate hydrogenlyase subunit/ One of the subunits of NADH:ubiquinone oxidoreductase
	acnA	1.7	Aconitate hydratase 1 /Antioxidant effect
	zwf	1.3	NADP(+)-dependent glucose-6-phosphate dehydrogenase/Produced NADPH, which maintain GSH levels in cells.
Stress response	marA	7.9	DNA-binding transcriptional dual regulator/ Regulate the transcription of various antibiotic and superoxide resistance genes.
	gshB	2.6	Glutathione synthetase/ Synthesized GSH (An important antioxidant)

^a^*FC* fold change

Knockout of *arcA* increased NADPH levels, whereas overexpression of *marA* resulted in decreased NADPH levels. Therefore, in theory, the effects of knocking out *arcA* and overexpressing *marA* are opposite, and the results of the combination of knocking out *arcA* and overexpressing *marA* need to be tested.

### Effects of promoters on the biocatalytic synthesis of cinnamylamine

The functional imbalance between ncCAR and OATA can lead to the accumulation of intermediates. In addition, high expression levels of enzymes may impose a burden on bacterial metabolism. Therefore, the purpose of this experiment is to optimize the promoters of the two enzymes to regulate the expression levels of the two enzymes and promote the functional balance of the two enzymes.

The intermediate cinnamaldehyde was accumulated in the whole-cell catalytic system, which indicated that the reaction catalyzed by OATA was a rate-limiting. The promoter T7 (P_T7_) of OATA belongs to the strong promoter, and theoretically, OATA has the high expression level. However, according to previous studies, a strong promoter is not necessarily required to express high levels of soluble protein [33]. Therefore, the P_T7_ of OATA was replaced with two weaker constitutive promoters P_1,6_ and P_2,51_ (Table S1). P_1,6_ and P_2,51_ are enhancing mutations of wild-type P_lac_, and the transcription intensity of P_1,6_ is 4 times that of P_2,51_ and 50 times that of P_lac_.

**Fig. 6:**
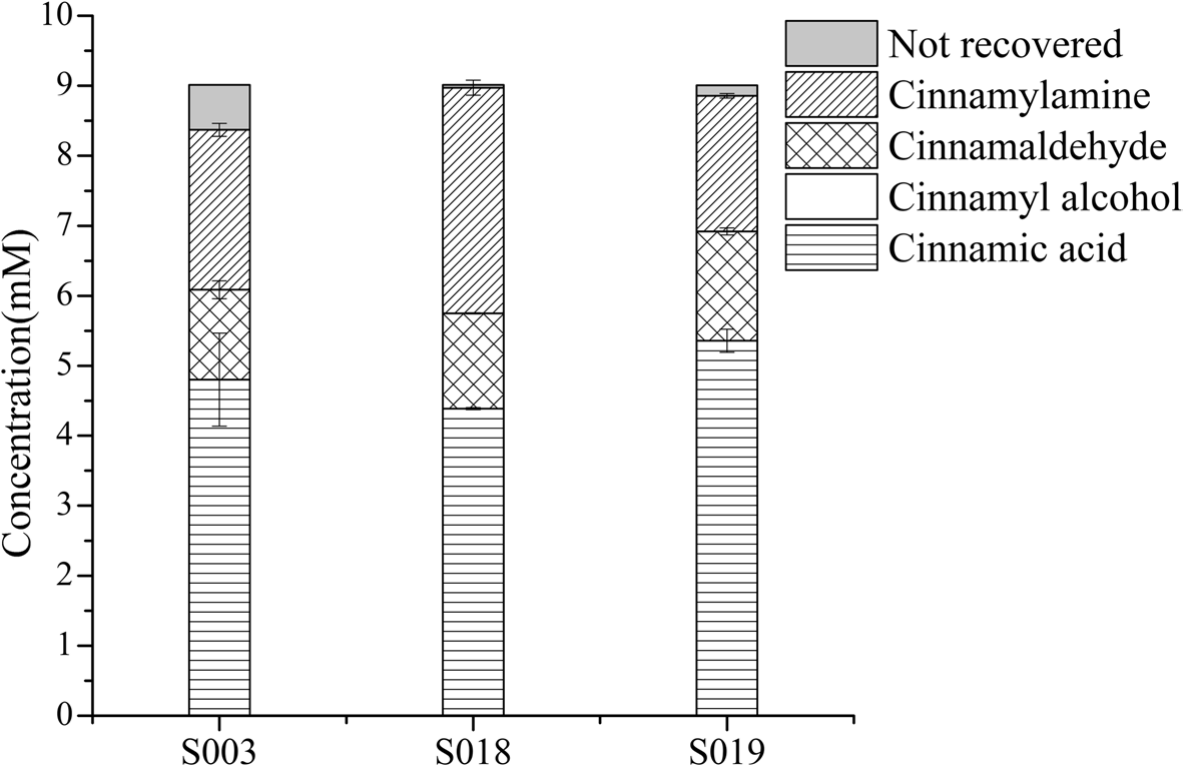
Effects of promoters on the yield of cinnamylamine and conversion of cinnamic acid. The reaction was performed in M9 buffer (pH 7.0) containing 9 mM cinnamic acid (the initial concentration); 20 mM L-Ala; 20 mM MgSO_4_; S003 or S018-S019 wet cells, OD_600 nm_ = 30 at 30 °C with 200 rpm shaking for 1 h

The promoter of OATA was replaced with P_1,6_ and P_2,51_ to obtain S018 and S019. Subsequently, the titers of cinnamylamine and cinnamylaldehyde of S003, S018 and S019 were detected. The results showed that compared with S003, the yield and productivity of cinnamylamine in S018 was increased by 80 and 43%, respectively. The yield of cinnamylamine in S019 was decreased (Fig. 6, Table 5).

**Table 5:** Effects of promoters on the yield, conversion, selectivity and productivity

Strains	Yield%	Conversion%	Selectivity %	Productivity (g/L/h)
S003	25.4	46.6	54.4	0.3 ± 0.012
S018	35.8	51.2	69.9	0.43 ± 0.014
S019	21.5	40.5	53.2	0.26 ± 0.004

The reaction conditions were the same as in Fig. 6

**Fig. 7:**
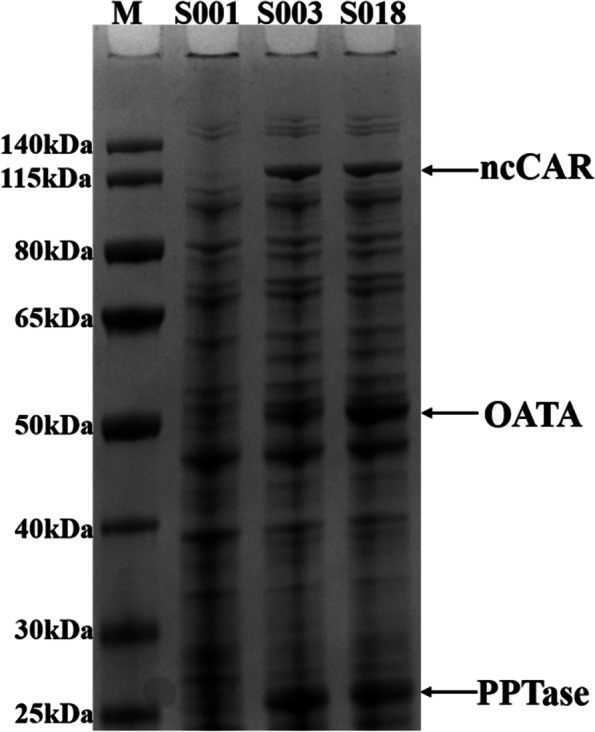
SDS-PAGE of S001, S003 and S018. M: Thermo Fisher 26,616 protein marker; S001: RARE(DE3); S003: RARE(DE3)/pET28a-*nccar-pptase*/pACYCDuet1-*oata*; S018: RARE(DE3)/pET28a-*nccar-pptase*/pACYCDuet1-P_1,6_-*oata*. The arrows indicated the location of the target protein

Whether the expression of soluble OATA increased after P_T7_ was replaced by P_1,6_ was analyzed by SDS-PAGE. The software Image Lab was used to analyze the expression level of soluble OATA. The content of OATA induced by P_T7_ accounted for 20.6% of the three target enzymes (ncCAR, OATA and PPTase), whereas the content of OATA induced by P_1,6_ accounted for 40.2% (Fig. 7). Therefore, P_1,6_-induced OATA is beneficial to increase the yield and productivity of cinnamylamine.

### Effect of combined positive factors on the biocatalytic synthesis of cinnamylamine

**Fig. 8:**
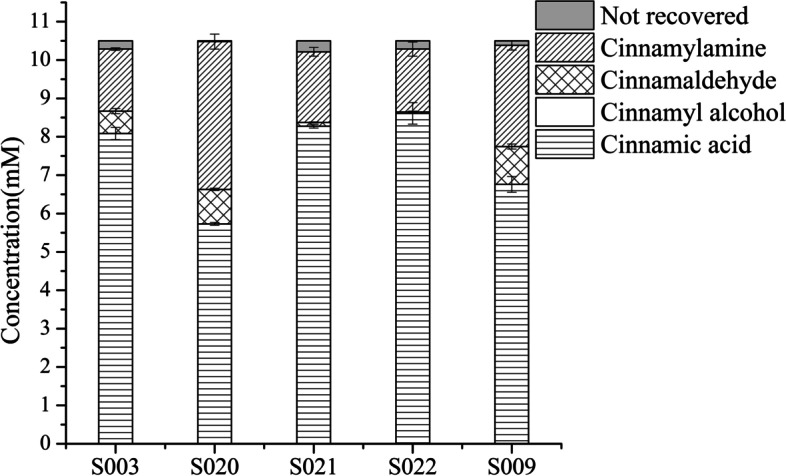
Effects of combined positive factors on the conversion of cinnamic acid to cinnamylamine. The reaction was performed in M9 buffer (pH 7.0) containing 10.5 mM cinnamic acid (the initial concentration); 20 mM L-Ala; 20 mM MgSO_4_; S003, S009 or S020-S022 wet cells, OD_600 nm_ = 30 at 30 °C with 200 rpm shaking for 1 h

The three positive factors screened above—knockout of *arcA*, overexpression of *marA* or replacement of P_T7_ with P_1,6_ in OATA, were combined to obtain S020-S022. The titers of cinnamylamine and cinnamaldehyde in S003, S009, S020, S021 and S022 were detected. The yield and productivity of cinnamylamine in S020 were the highest, which were 2.5 times and 2.4 times that of WT, respectively (Fig. 8). Although the accumulation of cinnamaldehyde increased by 54%, the selectivity of cinnamylamine increased overall. The yield of cinnamylamine in S021 increased by 15%, and the accumulation of cinnamaldehyde decreased by 76%. The yield of cinnamylamine in S022 remained unchanged, and the accumulation of cinnamaldehyde decreased by 94% (Table 6).

**Table 6:** Effects of combined positive factors on the yield, conversion, selectivity and productivity

Strains	Yield%	Conversion%	Selectivity %	Productivity (g/L/h)
S003	15.7	21.5	73	0.21 ± 0.004
S020	38.9	44.4	87.6	0.51 ± 0.026
S021	17.9	19.7	90.8	0.24 ± 0.015
S022	15.9	16.4	97	0.22 ± 0.024
S009	25.6	34.4	74.4	0.35 ± 0.016

The reaction conditions were the same as in Fig. 8

Compared with S020, the titers of cinnamylamine and cinnamylaldehyde were reduced in S022. This result indicated that the low NADPH level caused by overexpression of *marA* overshadowed the high NADPH level caused by the knockout of *arcA*, resulting in reduced accumulation of cinnamaldehyde and no residue, which resulted in a decrease in the titer of cinnamylamine as well. The titers of cinnamylamine and cinnamylaldehyde in S021 were lower than those of S009 for the same reason.

### Optimization of reaction conditions

**Fig. 9:**
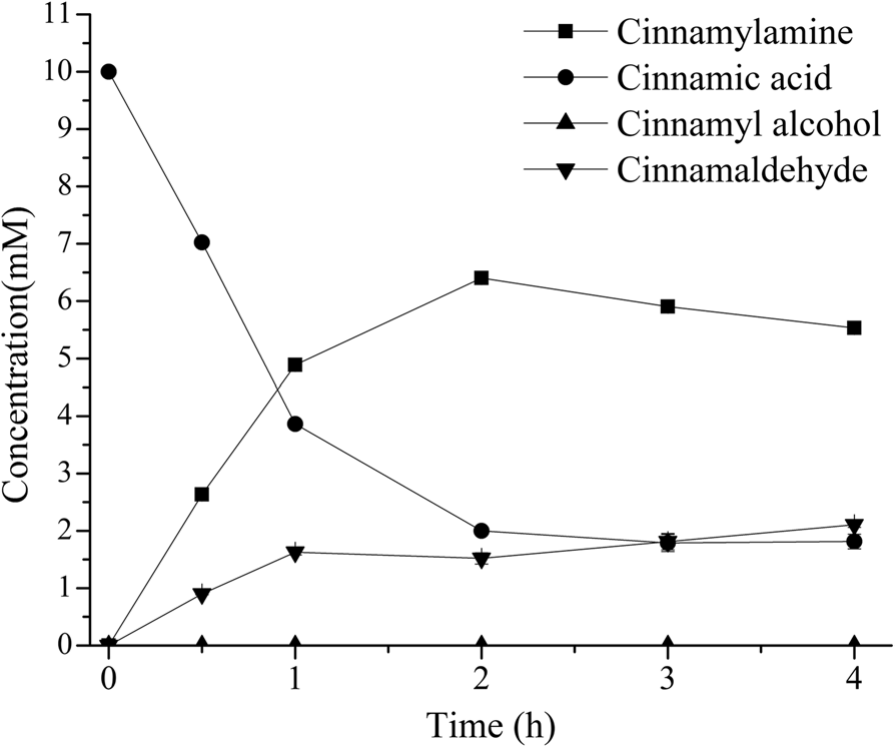
The effect of reaction time on the conversion of cinnamic acid to cinnamylamine. The reaction was performed in M9 buffer (pH 7.0) containing 10 mM cinnamic acid; 20 mM L-Ala; 20 mM MgSO_4_; S020 wet cells, OD_600 nm_ = 30 at 30 °C with 200 rpm shaking for 0.5, 1, 2, 3, 4 h

The strain S020 with the highest yield was obtained by metabolic regulation. In the next step, the yield and conversion are improved by optimization of the reaction conditions. Firstly, the reaction time is optimized. The yield of cinnamylamine and the conversion of cinnamic acid under different reaction times were detected. The results showed that the titer and yield of cinnamylamine in S020 reached the highest (6.4 mM, 64%) after 2 h (Fig. 9, Table 7). Thereafter, the yield of cinnamylamine decreased and the accumulation of cinnamaldehyde increased. There are two possible reasons: 1) The low solubility of cinnamylamine in water is not conducive to the conversion of cinnamaldehyde to cinnamylamine. It is necessary to increase the content of co-solvent DMSO in the system. 2) OATA is a reversible enzyme, and the co-substrate L-Ala will promote the reaction to proceed in the forward direction [34]. It is necessary to increase the L-Ala content in the system.

**Table 7:** The effect of reaction time on the yield, conversion, selectivity and productivity

Time (h)	Yield%	Conversion%	Selectivity %	Productivity (g/L/h)
1	48.9	61.4	79.6	0.65 ± 0.014
2	64	80	80	0.43 ± 0.002
3	59	82.1	71.9	0.26 ± 0.003
4	55.3	81.9	67.6	0.18 ± 0.002

The reaction conditions were the same as in Fig. 9

Next, the effect of DMSO content in the system on the yield and conversion was examined. The four systems contained 0, 5, 10, and 15% DMSO, respectively. The yield of cinnamylamine and the conversion of cinnamic acid in the four systems were detected. The results showed that the highest yield and conversion were achieved in the system containing 10% DMSO, which were 83.6 and 99.8%, respectively (Fig. S7 and Table S2). Then, the effect of L-Ala content on the yield and conversion was detected. Different concentrations of L-Ala (0, 10, 20 mM L-Ala) were added after 2 h. The yield of cinnamylamine and the conversion of cinnamic acid reached the highest after the addition of 10 mM L-Ala (Fig. S8 and Table S3).

**Fig. 10:**
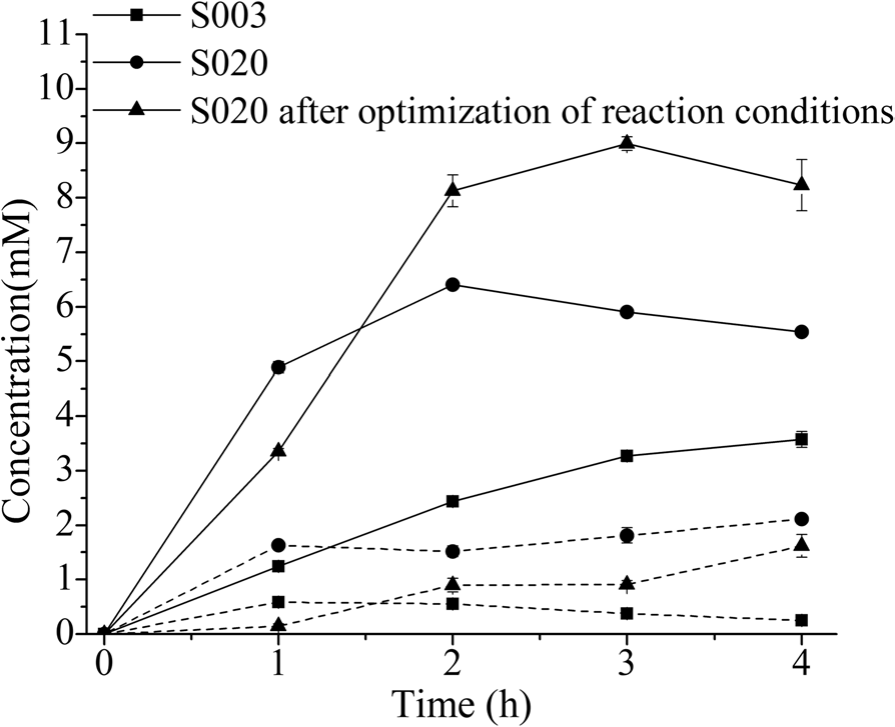
Time course of cinnamylamine production in S003, S020 and S020 after optimization of reaction conditions. Cinnamylamine concentration (solid lines) and cinnamaldehyde concentration (dashed lines) are demonstrated

Finally, the yields and conversions of the S003 and the S020 were compared. Before metabolic engineering, the yield of cinnamylamine and the conversion of cinnamic acid were very low (37 and 39%) after 4 h. After metabolic engineering, the yield of cinnamylamine was 2.5 times that of WT, and the conversion of cinnamic acid was 80%. However, there were still two problems, one was the accumulation of cinnamaldehyde (1.5 mM), and the other was that the yield and conversion no longer increase after 2 h. After optimization of the reaction conditions, the yield and conversion reached 90 and 100% after 3 h, respectively, and the accumulation of cinnamaldehyde was less than 0.9 mM. The productivity of cinnamylamine reached 0.4 g/L/h (Fig. 10, Table 8), and the production of cinnamylamine reached 1.2 g/L (9 mM). There were only two reports on the biosynthesis of cinnamylamine from cinnamic acid. One report from our lab reported that the production of cinnamylamine by whole-cell catalysis reached 4.2 mM (559 mg/L) [17]. Another report on the biosynthesis of cinnamylamine reported that the production of cinnamylamine by shaking flask culture reached 523 mg/L [35]. Therefore, 1.2 g/L was the highest production of biosynthetic cinnamylamine.

**Table 8:** The effect of S003, S020 and the optimized S020 on the yield, conversion, selectivity and productivity

No.	Time (h)	Yield%	Conversion%	Selectivity %	Productivity (g/L/h)
S003	1	12	18.7	64.3	0.17 ± 0.006
	2	23.4	28.3	82.6	0.16 ± 0.004
	3	29.9	33.5	89.2	0.14 ± 0.004
	4	34.3	35.1	97.8	0.12 ± 0.005
S020	1	48.9	61.4	79.6	0.65 ± 0.014
	2	64	80	80	0.43 ± 0.002
	3	59	82.1	71.9	0.26 ± 0.003
	4	55.3	81.9	67.6	0.18 ± 0.002
optimized S020	1	33.5	33.4	100	0.45 ± 0.007
	2	81.3	86.4	94	0.54 ± 0.019
	3	90	100	90	0.4 ± 0.010
	4	83.9	100	83.9	0.28 ± 0.003

## Conclusion

APAs are key intermediates in the chemical industry with extensive applications in the manufacture of pharmaceuticals, pesticides, polymers, dyes and detergents. At present, the accumulation of toxic intermediates (aldehydes) during biosynthesis affects yields of APAs due to metabolic imbalance.

In this work, the biocatalytic synthesis of APAs (taking cinnamylamine as an example) was metabolically regulated by the overexpression or knockout of five native global TFs, eight native resistance genes and optimization of promoters. The global TF *arcA* was knocked out and the P_T7_ of OATA was replaced by P_1,6_ in the optimized metabolically engineered strain S020. The yield of S020 was 2.5 times that of S003 (WT). After optimization of the reaction conditions, the yield of cinnamylamine and the conversion of cinnamic acid in S020 reached 90 and 100%, respectively, and the accumulation of cinnamaldehyde was less than 0.9 mM. Finally, the bottleneck of low yield of cinnamylamine and high accumulation of cinnamaldehyde was solved.

In addition, the reasons for the increased yield of cinnamylamine and the decreased accumulation of cinnamaldehyde in S020 were analyzed. The results show that knocking out *arcA* can increase the intracellular levels of NADPH and ATP and accelerate the metabolism of the by-product pyruvate, which is beneficial for the conversion of cinnamic acid to cinnamylamine. After the P_T7_ of OATA was replaced by P_1,6_, the expression level of OATA was significantly increased, which was favorable for the conversion of cinnamaldehyde to cinnamylamine.

Previously, our lab has reported that this pathway is also suitable for the biosynthesis of other APAs. Since the accumulation of NADPH and ATP after the knockout of *arcA* and the improvement of the expression level of OATA after the replacement of the promoter belong to the changes of the strain itself, this metabolic regulatory process is theoretically suitable for regulating the biosynthesis of other APAs. In other words, this metabolic regulation process provides a common strategy for the efficient synthesis of other APAs.

## Supplementary Information

**Additional file 1: Fig. S1.** Chemical synthesis routes of several APAs. **Fig. S2.** Volcano plot of DEGs between S003 and S009. **Fig. S3.** DEGs in TCA cycle pathway between S003 and S009. **Fig. S4.** DEGs in oxidative phosphorylation pathway between S003 and S009. **Fig. S5.** DEGs in pyruvate metabolism pathway between S003 and S009. **Fig. S6.** Volcano plot of DEGs between S003 and S010. **Fig. S7.** The effect of DMSO on the conversion of cinnamic acid to cinnamylamine by ncCAR and OATA. **Fig. S8.** The effect of L-Ala on the conversion of cinnamic acid to cinnamylamine by ncCAR and OATA. **Table S1.** Sequences of P1,6 and P2,51 promoter. **Table S2.** The effect of DMSO on yield, conversion and selectivity. **Table S3.** The effect of L-Ala on yield, conversion and selectivity.

## Data Availability

All relevant data will be freely available post-publication to any scientist that show interest or made a request.

## Abbreviations

APAs: Aromatic primary amines
ncCAR: Carboxylic acid reductase from *Neurospora crassa*
OATA: ω-transaminase from *Ochrobactrum anthropi*
PPTase: Phosphopantetheine transferase from *E. coli*
RARE (DE3): MG1655(DE3) *∆dkgB ∆yeaE ∆(yqhC-dkgA) ∆yahK ∆yjgB ∆yqhD*
WT: Wild-type strain
L-Ala: L-alanine
TFs: Transcription factors
DEGs: Differentially expressed genes
MDTs: Multidrug resistance transporters
IPTG: Isopropyl beta-D-1-thiogalactopyranoside
Kan: Kanamycin
Cm: Chloramphenicol
FC: Fold change
SDS-PAGE: Sodium dodecyl sulfate polyacrylamide gel electrophoresis

## References

[1] Adam R, Bheeter CB, Cabrero-Antonino JR, Junge K, Jackstell R, Beller M (2017). Selective hydrogenation of nitriles to primary amines by using a cobalt phosphine catalyst. ChemSusChem.

[2] Liang G, Wang A, Li L, Chemie DJA (2017). Production of primary amines by reductive amination of biomass-derived aldehydes/ketones.

[3] Chao X, Jinliang S, Manli H, Yue H, Buxing H (2020). Ambient-temperature synthesis of primary amines via reductive amination of carbonyl compounds. ACS Catal.

[4] Yufei S, Wenhu L, Shangpeng F (2014). Preparation and application of 3-AMPR (aminomethylpyridine resin)-1,3-propane sultone-acid radical anion catalyst. CN104475154B.

[5] Yun WJ, Ching CT, Shiou ZM (2013). Influence of Counteranions on the structural modulation of silver–Di(3-pyridylmethyl)amine coordination polymers. Cryst Growth Des.

[6] Jing J, Huo X, Shen J, Fu J, Meng Q, Zhang W (2017). Direct use of allylic alcohols and allylic amines in palladium-catalyzed allylic amination. Chem Commun (Camb).

[7] Zhang D, Song X, Su J (2014). Isolation, identification and characterization of novel process-related impurities in flupirtine maleate. J Pharm Biomed Anal.

[8] Wang Z, Wang Y, Pasangulapati JP, Stover KR, Liu X, Schier SW, Weave DF (2021). Design, synthesis, and biological evaluation of furosemide analogs as therapeutics for the proteopathy and immunopathy of Alzheimer's disease. Eur J Med Chem.

[9] Xianheng W, Song C, Changkuo Z, Liangye L, Yuhe W (2019). Preparation of Dolutegravir intermediate Diastereomer. J Heterocyclic Chem.

[10] Ferraccioli R, Borovika D, Surkus A-E, Kreyenschulte C, Topf C, Beller M (2018). Synthesis of cobalt nanoparticles by pyrolysis of vitamin B12: a non-noble-metal catalyst for efficient hydrogenation of nitriles. Catal Sci Technol.

[11] Segobia DJ, Trasarti AF, Apesteguía CR (2015). Chemoselective hydrogenation of unsaturated nitriles to unsaturated primary amines: conversion of cinnamonitrile on metal-supported catalysts. Appl Catal A Gen.

[12] Maela M, Calcio GE, Giancarlo C, Silvia T, Nasir BRB, Evelina C, Varma RS (2019). Microwave-assisted reductive amination with aqueous Ammonia: sustainable pathway using recyclable magnetic nickel-based Nanocatalyst. ACS Sustain Chem Eng.

[13] Duo TC, Ju DP, Ling SH, Yuan JY, Zhi ZM (2019). One-pot synthesis of Phenylglyoxylic acid from racemic Mandelic acids via Cascade biocatalysis. J Agric Food Chem.

[14] Shuke W, Zhi L (2018). Whole-cell Cascade biotransformations for one-pot multistep organic synthesis. ChemCatChem.

[15] Yufen C, Peiling W, Liangyu K, Tzuyu K, Lijun L, Yang Z, Jifeng Y (2020). High-yielding Protocatechuic acid synthesis from l-tyrosine in Escherichia coli. ACS Sustain Chem Eng.

[16] Zhongwei Z, Qian L, Fei W, Renjie L, Xiaojuan Y, Lixin K, Jing Z, Aitao L (2020). One-pot biosynthesis of 1,6-hexanediol from cyclohexane byde novodesigned cascade biocatalysis. Green Chem.

[17] Shan Y, Miaomiao J, Chao X, Wencheng Y, Mingsha Z, Mo X, et al. Self-sufficient whole-cell biocatalysis for 3-(aminomethyl) pyridine synthesis. Biochem Eng J. 2022:183. 10.1016/j.bej.2022.108457.

[18] Schwendenwein D, Fiume G, Weber H, Rudroff F, Winkler M (2016). Selective enzymatic transformation to aldehydes in vivo by fungal carboxylate reductase from Neurospora crassa. Adv Synth Catal.

[19] Bang HB, Lee YH, Kim SC, Sung CK, Jeong KJ. Metabolic engineering of Escherichia coli for the production of cinnamaldehyde. Microb Cell Factories. 2016:15. 10.1186/s12934-016-0415-9. 10.1186/s12934-016-0415-9 PMC4719340 26785776

[20] Zhou Y, Mao SWJ, Li Z (2018). Bioproduction of Benzylamine from renewable feedstocks via a nine-step artificial enzyme Cascade and engineered metabolic pathways. ChemSusChem.

[21] Liu C, Ding Y, Zhang R, Liu H, Xian M, Zhao G (2016). Functional balance between enzymes in malonyl-CoA pathway for 3-hydroxypropionate biosynthesis. Metab Eng.

[22] Deng C, Lv X, Jianghua, Zhang H, Liu Y, Du G, Amaro RL, Liu L (2021). Synergistic improvement of N-acetylglucosamine production by engineering transcription factors and balancing redox cofactors. Metab Eng.

[23] Kunjapur AM, Tarasova Y, Prather KLJ (2014). Synthesis and accumulation of aromatic aldehydes in an engineered strain of Escherichia coli. J Am Chem Soc.

[24] Liu M, Fu Y, Gao W, Xian M, Zhao G (2020). Highly efficient biosynthesis of hypoxanthine in Escherichia coli and transcriptome-based analysis of the purine metabolism. ACS Synth Biol.

[25] Tomoya B, Takeshi A, Miki H, Yuki T, Yoshiko O, Miki B, Datsenko KA, Masaru T, Wanner LB, Hirotada M (2006). Construction of Escherichia coli K-12 in-frame, single-gene knockout mutants: the Keio collection. Mol Syst Biol.

[26] Matsuoka Y, Kurata H (2017). Modeling and simulation of the redox regulation of the metabolism in Escherichia coli at different oxygen concentrations. Biotechnol Biofuels.

[27] Lu XY, Ren SL, Lu JZ, Zong H, Song J, Zhuge B (2018). Enhanced 1,3-propanediol production in Klebsiella pneumoniae by a combined strategy of strengthening the TCA cycle and weakening the glucose effect. J Appl Microbiol.

[28] Scheel RA, Ji L, Lundgren BR, Nomura CT (2016). Enhancing poly(3-hydroxyalkanoate) production in Escherichia coli by the removal of the regulatory gene arcA. AMB Express.

[29] Liu M, Yao L, Xian M, Ding Y, Liu H, Zhao G (2016). Deletion of arcA increased the production of acetyl-CoA-derived chemicals in recombinant Escherichia coli. Biotechnol Lett.

[30] Wang Y, Xian M, Feng X, Liu M, Zhao G (2018). Biosynthesis of ethylene glycol from d-xylose in recombinant Escherichia coli. Bioengineered.

[31] Feifei H, Mo X, Wei H (2021). De novo biosynthesis and whole-cell catalytic production of paracetamol on a gram scale in Escherichia coli. Green Chem.

[32] Visvalingam J, Hernandez-Doria JD, Holley RA (2013). Examination of the genome-wide transcriptional response of Escherichia coli O157:H7 to cinnamaldehyde exposure. Appl Environ Microbiol.

[33] Shan Y, Rubing Z, Yujin C, Jing G, Mo X, Wei L (2020). New expression system to increase the yield of phloroglucinol. Biotechnol Biotechnol Equip.

[34] Han SW, Kim J, Cho HS, Shin JS (2017). Active site engineering of ω-transaminase guided by docking orientation analysis and virtual activity screening. ACS Catal.

[35] Wang Q, Ma L, Wang Z, Chen Q, Wang Q, Qi Q (2022). Construction and yield optimization of a cinnamylamine biosynthesis route in Escherichia coli. Biotechnol Biofuels Bioprod.

