# Supplementary material for: Synergistic improvement of cinnamylamine production by metabolic regulation
Source: J Biol Eng. 2023 Feb 23;17:14. doi: 10.1186/s13036-023-00334-y (PMC9948449; doi:10.1186/s13036-023-00334-y)
Supplement: Supplementary file 1 — Additional file 1: Fig. S1. Chemical synthesis routes of several APAs. Fig. S2. Volcano plot of DEGs between S003 and S009. Fig. S3. DEGs in TCA cycle pathway between S003 and S009. Fig. S4. DEGs in oxidative phosphorylation pathway between S003 and S009. Fig. S5. DEGs in pyruvate metabolism pathway between S003 and S009. Fig. S6. Volcano plot of DEGs between S003 and S010. Fig. S7. The effect of DMSO on the conversion of cinnamic acid to cinnamylamine by ncCAR and OATA. Fig. S8. The effect of L-Ala on the conversion of cinnamic acid to cinnamylamine by ncCAR and OATA. Table S1. Sequences of P1,6 and P2,51 promoter. Table S2. The effect of DMSO on yield, conversion and selectivity. Table S3. The effect of L-Ala on yield, conversion and selectivity. [file 13036_2023_334_MOESM1_ESM.zip › Revised supplement information-2.docx]

# Synergistic improvement of cinnamylamine production by metabolic regulation

Shan Yuan ^a, b^, Chao Xu ^a^, Miaomiao Jin ^a^, Mo Xian ^a^* & Wei Liu ^a^*

*Corresponding authors. E-mail addresses: xianmo@qibebt.ac.cn; liuwei@qibebt.ac.cn.

^a^ CAS Key Laboratory of Biobased Materials, Qingdao Institute of Bioenergy and Bioprocess Technology, Chinese Academy of Sciences, No. 189 Songling Road, Qingdao, 266101, Shandong, PR China; ^b^ University of Chinese Academy of Sciences, Beijing, PR China.

## 1. Supplementary methods for transcriptome analysis

### 1.1 Sample collection and preparation

#### 1.1.1 RNA quantification and qualification

Total RNA was extracted from the *E. coli* S003, S009 and S010 using RNeasy Mini kit (Qiagen, Germany) following the manufacturer’s protocol. The experiment was done in duplicate. RNA concentration was measured using NanoDrop 2000 (Thermo, USA). RNA integrity was assessed using the RNA Nano 6000 Assay Kit of the Agilent Bioanalyzer 2100 system (Agilent Technologies, USA).

#### 1.1.2 Library preparation for Transcriptome sequencing

A total amount of 1 μg RNA per sample was used as input material for the RNA sample preparations. Sequencing libraries were generated using NEBNext®Ultra™ RNA Library Prep Kit for Illumina® (NEB, USA) following manufacturer’s recommendations and index codes were added to attribute sequences to each sample. Briefly, mRNA was purified from total RNA using poly-T oligo-attached magnetic beads. Fragmentation was carried out using divalent cations under elevated temperature in NEBNext First Strand Synthesis Reaction Buffer (5X). First strand cDNA was synthesized using random hexamer primer and M-MuLV Reverse Transcriptase. Second strand cDNA synthesis was subsequently performed using DNA Polymerase I and RNase H. Remaining overhangs were converted into blunt ends via exonuclease/polymerase activities. After adenylation of 3’ ends of DNA fragments, NEBNext Adaptor with hairpin loop structure were ligated to prepare for hybridization. In order to select cDNA fragments of preferentially 240 bp in length, the library fragments were purified with AMPure XP system (Beckman Coulter, USA). Then 3 μL USER Enzyme (NEB, USA) was used with size-selected, adaptor-ligated cDNA at 37 °C for 15 min followed by 5 min at 95 °C before PCR. Then PCR was performed with Phusion High-Fidelity DNA polymerase, Universal PCR primers and Index (X) Primer. At last, PCR products were purified and library quality was assessed on the Agilent Bioanalyzer 2100 system.

#### 1.1.3 Clustering and sequencing

The clustering of the index-coded samples was performed on a cBot Cluster Generation System using TruSeq PE Cluster Kit v3-cBot-HS (Illumia, USA) according to the manufacturer’s instructions. After cluster generation, the library preparations were sequenced on an Illumina Hiseq platform and paired-end reads were generated.

### 1.2 Data analysis

#### 1.2.1 Quality control

Raw data (raw reads) of fastq format were firstly processed through in-house perl scripts. In this step, clean data(clean reads) were obtained by removing reads containing adapter, reads containing ploy-N and low quality reads from raw data. At the same time, Q20, Q30, GC-content and sequence duplication level of the clean data were calculated. All the downstream analyses were based on clean data with high quality.

#### 1.2.2 Comparative analysis

The adaptor sequences and low-quality sequence reads were removed from the data sets. Raw sequences were transformed into clean reads after data processing. These clean reads were then mapped to the reference genome sequence. Only reads with a perfect match or one mismatch were further analyzed and annotated based on the reference genome. Tophat2 tools soft were used to map with reference genome.

#### 1.2.3 Gene functional annotation

Gene function was annotated based on the following databases: Nr (NCBI non-redundant protein sequences)；Nt (NCBI non-redundant nucleotide sequences)；Pfam (Protein family)；KOG/COG (Clusters of Orthologous Groups of proteins)；Swiss-Prot (A manually annotated and reviewed protein sequence database)；KO (KEGG Ortholog database)；GO (Gene Ontology).

#### 1.2.4 SNP calling

Picard-tools v1.41 and samtools v0.1.18 were used to sort, remove duplicated reads and merge the bam alignment results of each sample. GATK2 or Samtools software was used to perform SNP calling. Raw vcffiles were filtered with GATK standard filter method and other parameters ( clusterWindowSize: 10; MQ0 >= 4 and (MQ0/(1.0*DP)) > 0.1; QUAL < 10; QUAL < 30.0 or QD < 5.0 or HRun > 5), and only SNPs with distance > 5 were retained.

#### 1.2.5 Quantification of gene expression levels

Quantification of gene expression levels were estimated by fragments per kilobase of transcript per million fragments mapped (FPKM). The formula is shown as follow:

$$FPKM=cDNA Fragments\div\left[ Mapped Fragments \left( Millions \right)\times Transcript Length \left( kb \right) \right]$$

#### 1.2.6 Differential expression analysis

Differential expression analysis of two groups was performed using the DESeq R package (1.10.1). DESeq provide statistical routines for determining differential expression in digital gene expression data using a model based on the negative binomial distribution. The resulting P values were adjusted using the Benjamini and Hochberg’s approach for controlling the false discovery rate. Genes with P-value < 0.05 and |log_2_(foldchange)| ≥1 were assigned as differentially expressed.

#### 1.2.7 GO enrichment analysis

Gene Ontology (GO) enrichment analysis of the differentially expressed genes (DEGs) was implemented by the GOseq R packages based Wallenius non-central hyper-geometric distribution ^1^, which can adjust for gene length bias in DEGs.

#### 1.2.8 KEGG pathway enrichment analysis

KEGG ^2^ is a database resource for understanding high-level functions and utilities of the biological system, such as the cell, the organism and the ecosystem, from molecular-level information, especially large-scale molecular datasets generated by genome sequencing and other high-throughput experimental technologies (http://www.genome.jp/kegg/). We used KOBAS ^3^ software to test the statistical enrichment of differential expression genes in KEGG pathways.

#### 1.2.9 PPI (Protein Protein Interaction)

The sequences of the DEGs was blast (blastx) to the genome of a related species (the protein protein interaction of which exists in the STRING database: http://string-db.org/) to get the predicted PPI of these DEGs. Then the PPI of these DEGs were visualized in Cytoscape ^4^.

## 2. Contents of supplement figures and tables

Fig. S1 Chemical synthesis routes of several APAs.

Fig. S2 Volcano plot of DEGs between S003 and S009.

Fig S3 DEGs in TCA cycle pathway between S003 and S009.

Fig S4 DEGs in oxidative phosphorylation pathway between S003 and S009.

Fig S5 DEGs in pyruvate metabolism pathway between S003 and S009.

Fig. S6 Volcano plot of DEGs between S003 and S010.

Fig. S7 The effect of DMSO on the conversion of cinnamic acid to cinnamylamine by ncCAR and OATA.

Fig. S8 The effect of L-Ala on the conversion of cinnamic acid to cinnamylamine by ncCAR and OATA.

Table S1 Sequences of P_1,6_ and P_2,51_ promoter.

Table S2 The effect of DMSO on yield, conversion and selectivity.

Table S3 The effect of L-Ala on yield, conversion and selectivity.

Fig. S1 Chemical synthesis routes of several APAs.


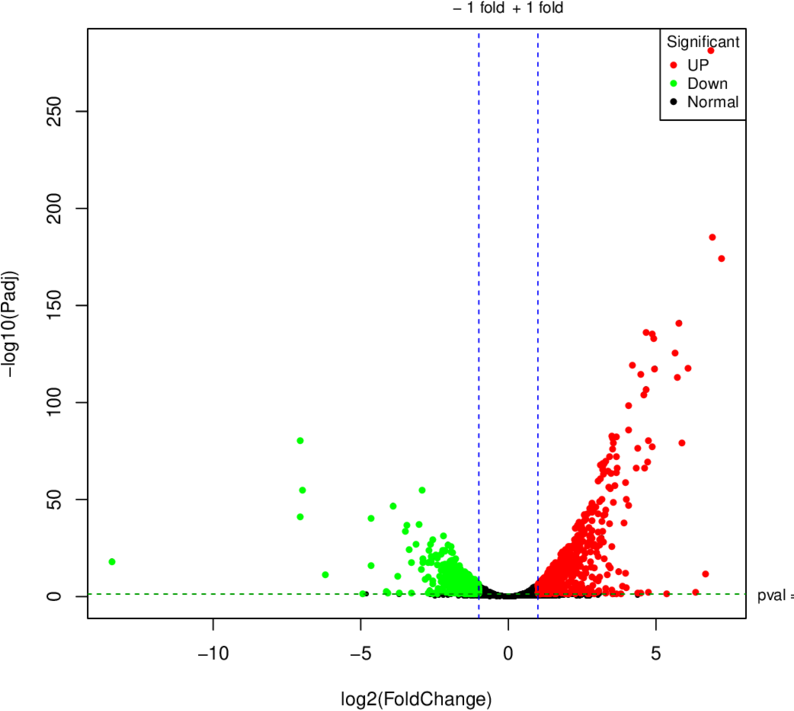


Fig. S2 Volcano plot of DEGs between S003 and S009.

Each point in the volcano plot represents a gene. The abscissa represents log_2_ (fold change). The fold change represents the fold change of the expression level of a gene in two samples. The vertical axis represents -log_10_ (Padj), and -log_10_ (Padj) represents significance. The greater the |log_2_ (fold change)|, the greater the difference in the expression level of a gene between the two samples. The larger the -log_10_ (Padj) of a gene, the more reliable the DEGs obtained by screening.


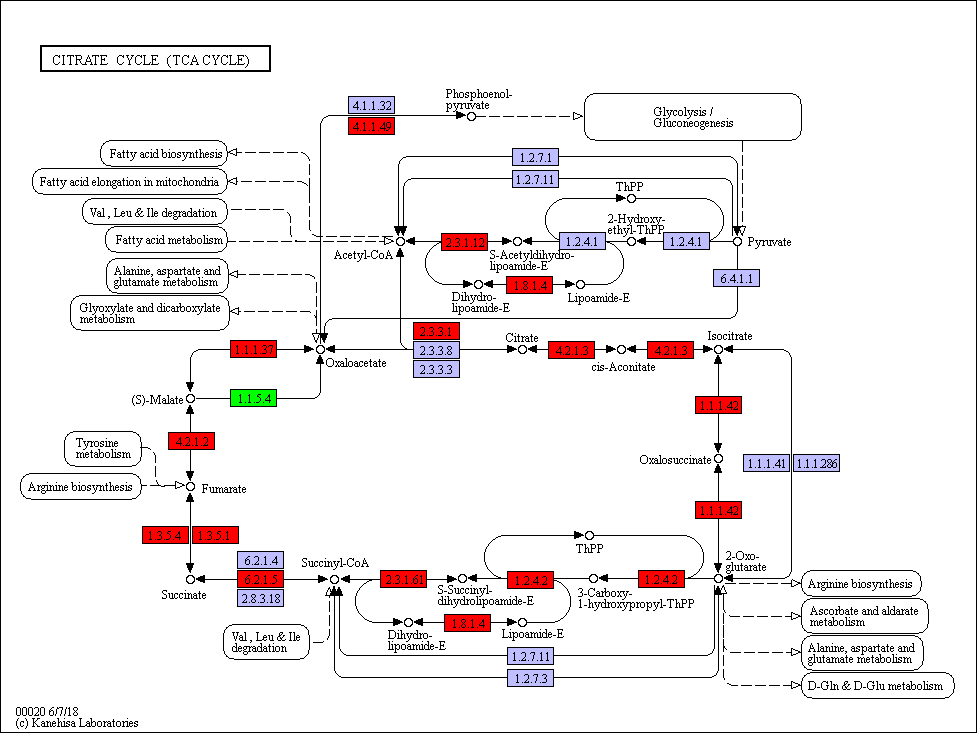


Fig S3 DEGs in TCA cycle pathway between S003 and S009.

The EC numbers of up-regulated genes (log_2_ fold change>1) are marked in red and the EC numbers of down-regulated genes (log_2_ fold change>-1) are marked in green. When up-regulated genes and down-regulated genes belong to the same EC number, they are marked in yellow.


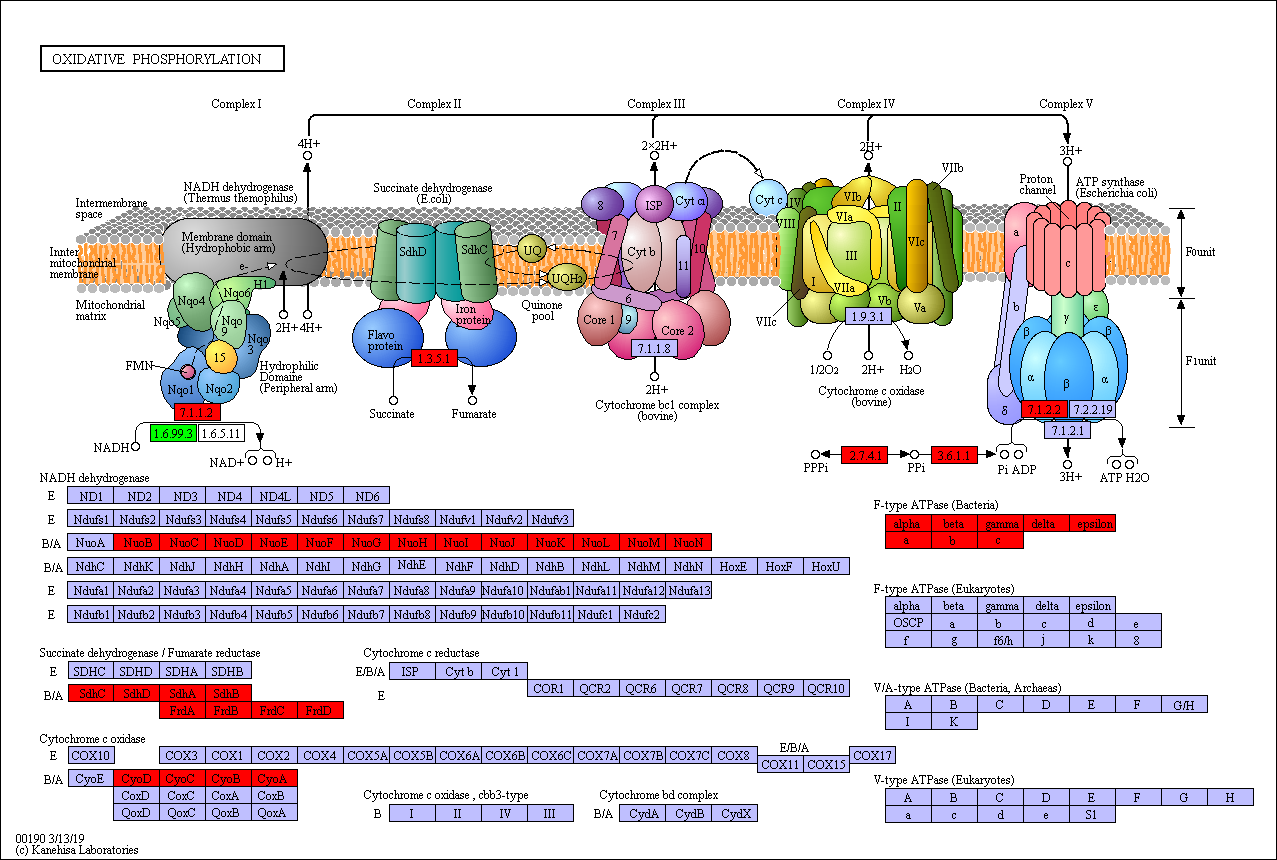


Fig S4 DEGs in oxidative phosphorylation pathway between S003 and S009.


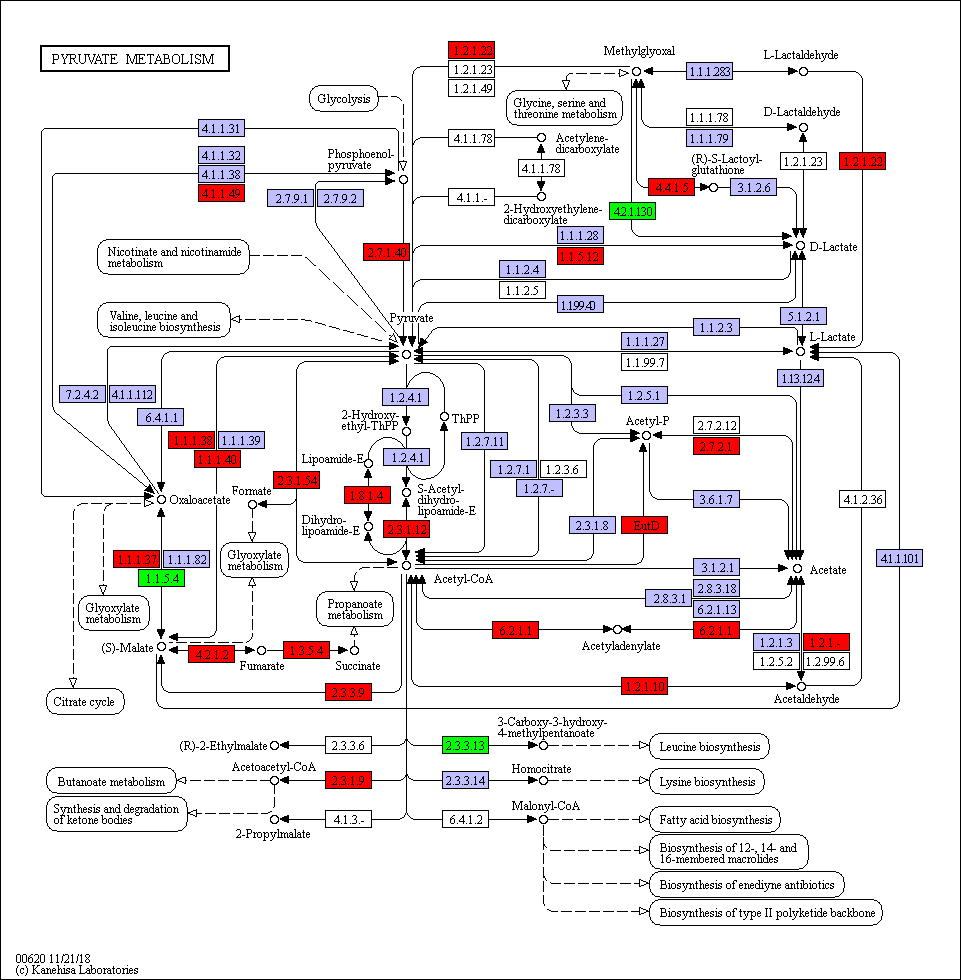


Fig S5 DEGs in pyruvate metabolism pathway between S003 and S009.


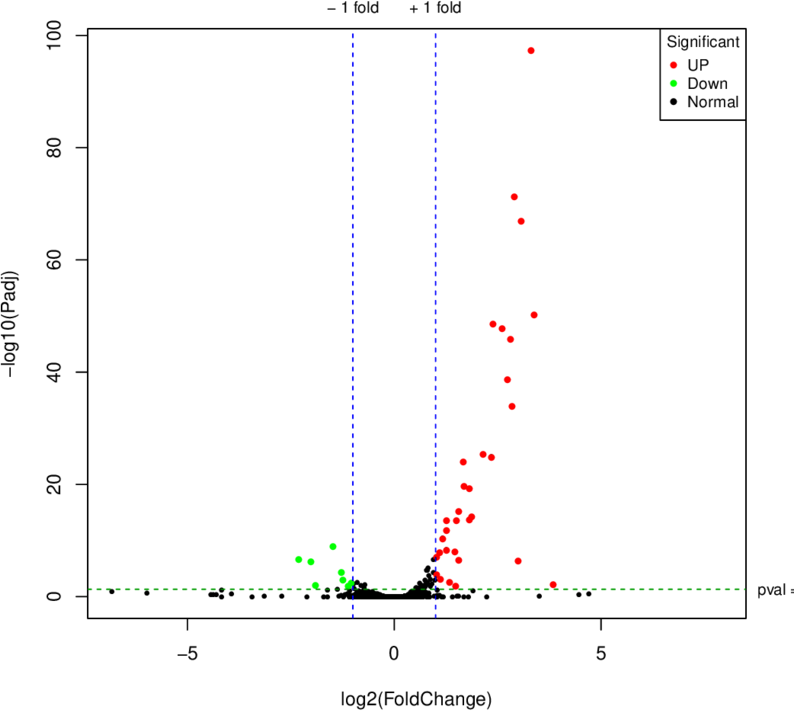


Fig. S6 Volcano plot of DEGs between S003 and S010.





Fig. S7 The effect of DMSO on the conversion of cinnamic acid to cinnamylamine by ncCAR and OATA.

Cinnamylamine concentration (solid lines) and cinnamaldehyde concentration (dashed lines) are demonstrated. The reaction was performed in M9 buffer (pH 7.0) containing 10.5 mM cinnamic acid; 20 mM L-Ala; 20 mM MgSO_4_; 0%-15% DMSO; S020 wet cells, OD_600 nm_= 30 at 30 ^◦^C with 200 rpm shaking for 1, 2, 3, 4 h.





Fig. S8 The effect of L-Ala on the conversion of cinnamic acid to cinnamylamine by ncCAR and OATA.

The reaction was performed in M9 buffer (pH 7.0) containing 10 mM cinnamic acid; 20 mM L-Ala; 20 mM MgSO_4_; 10%DMSO; S020 wet cells, OD_600 nm_= 30 at 30 ^◦^C with 200 rpm shaking for 1, 2, 3, 4 h. Additional 0, 10, 20 mM L-Ala was added after 2 h.

Table S1 Sequences of P_1,6_ and P_2,51_ promoter

| Promoter | Promoter Sequence (5’- 3’) |
| --- | --- |
| P_1,6_ | CTTTACACTTTAAGCTTTTTATGTTTATGTTGTGTGGAA |
| P_2,51_ | CTTTACACTTTAAGCTTCATATGTTTATGTTGTGTGGAA |

Table S2 The effect of DMSO on yield, conversion and selectivity.

| Time(h) | 0%DMSO | | | 5%DMSO | | | 10%DMSO | | | 15%DMSO | | | |
| --- | --- | --- | --- | --- | --- | --- | --- | --- | --- | --- | --- | --- | --- |
| --- | Y% | C% | S% | Y% | C% | S% | Y% | C% | S% | Y% | C% | S% |  |
| 1 | 47.4 | 55.6 | 85.3 | 54.5 | 61.9 | 88 | 53.8 | 57.2 | 94 | 32.1 | 30.2 | 100 |  |
| 2 | 66.3 | 74.9 | 88.6 | 73.9 | 85.1 | 86.9 | 77.6 | 85.7 | 90.5 | 46.7 | 44.2 | 100 |  |
| 3 | 62.7 | 79.7 | 78.6 | 73.2 | 93 | 78.8 | 83.6 | 99.8 | 83.7 | 61.4 | 59.8 | 100 |  |
| 4 | 58.4 | 79.2 | 73.7 | 68.9 | 92.9 | 74.2 | 79.9 | 100 | 79.9 | 68.4 | 66.8 | 100 |  |

Y%: yield%; C%: conversion%; S%: selectivity%.

The reaction was performed in M9 buffer (pH 7.0) containing 10.5 mM cinnamic acid; 20 mM L-Ala; 20 mM MgSO_4_; 0%-15% DMSO; S020 wet cells, OD_600 nm_= 30 at 30 ^◦^C with 200 rpm shaking for 1, 2, 3, 4 h.

Table S3 The effect of L-Ala on yield, conversion and selectivity.

| Time(h) | 0 mM L-Ala | | | 10 mM L-Ala | | | 20 mM L-Ala | | |
| --- | --- | --- | --- | --- | --- | --- | --- | --- | --- |
| --- | Y% | C% | S% | Y% | C% | S% | Y% | C% | S% |
| 1 | 33.5 | 33.4 | 100 | 33.5 | 33.4 | 100 | 33.5 | 33.4 | 100 |
| 2 | 81.3 | 86.4 | 94 | 81.3 | 86.4 | 94 | 81.3 | 86.4 | 94 |
| 3 | 87.3 | 100 | 87.3 | 90 | 100 | 90 | 89.9 | 100 | 89.9 |
| 4 | 83.3 | 100 | 83.3 | 83.9 | 100 | 83.9 | 82.3 | 100 | 82.3 |

Y%: yield%; C%: conversion%; S%: selectivity%.

The reaction was performed in M9 buffer (pH 7.0) containing 10 mM cinnamic acid; 20 mM L-Ala; 20 mM MgSO_4_; 10% DMSO; S020 wet cells, OD_600 nm_= 30 at 30 ^◦^C with 200 rpm shaking for 1, 2, 3, 4 h. Additional 0, 10, 20 mM L-Ala was added after 2 h.

## References

(1) Young, M. D.; Wakefield, M. J.; Smyth, G. K.; Oshlack, A., Gene ontology analysis for RNA-seq accounting for selection bias. *Genome biology* **2010,** *11*, R14. <http://doi.org/10.1186/gb-2010-11-2-r14>.

(2) Kanehisa, M.; Araki, M.; Goto, S.; Hattori, M.; Hirakawa, M.; Itoh, M.; Katayama, T.; Kawashima, S.; Okuda, S.; Tokimatsu, T.; Yamanishi, Y., KEGG for linking genomes to life and the environment. *Nucleic Acids Res* **2008,** *36*, D480-484. <http://doi.org/10.1093/nar/gkm882>.

(3) Mao, X.; Cai, T.; Olyarchuk, J. G.; Wei, L., Automated genome annotation and pathway identification using the KEGG Orthology (KO) as a controlled vocabulary. *Bioinformatics* **2005,** *21*, 3787-3793. <https://doi.org/10.1093/bioinformatics/bti430>.

(4) Shannon, P.; Markiel, A.; Ozier, O.; Baliga, N. S.; Wang, J. T.; Ramage, D.; Amin, N.; Schwikowski, B.; Ideker, T., Cytoscape: a software environment for integrated models of biomolecular interaction networks. *Genome Res* **2003,** *13*, 2498-2504. <http://doi.org/10.1101/gr.1239303>.
