# Supplementary figures and images for: Synergistic improvement of cinnamylamine production by metabolic regulation
Source: J Biol Eng. 2023 Feb 23;17:14. doi: 10.1186/s13036-023-00334-y (PMC9948449; doi:10.1186/s13036-023-00334-y)

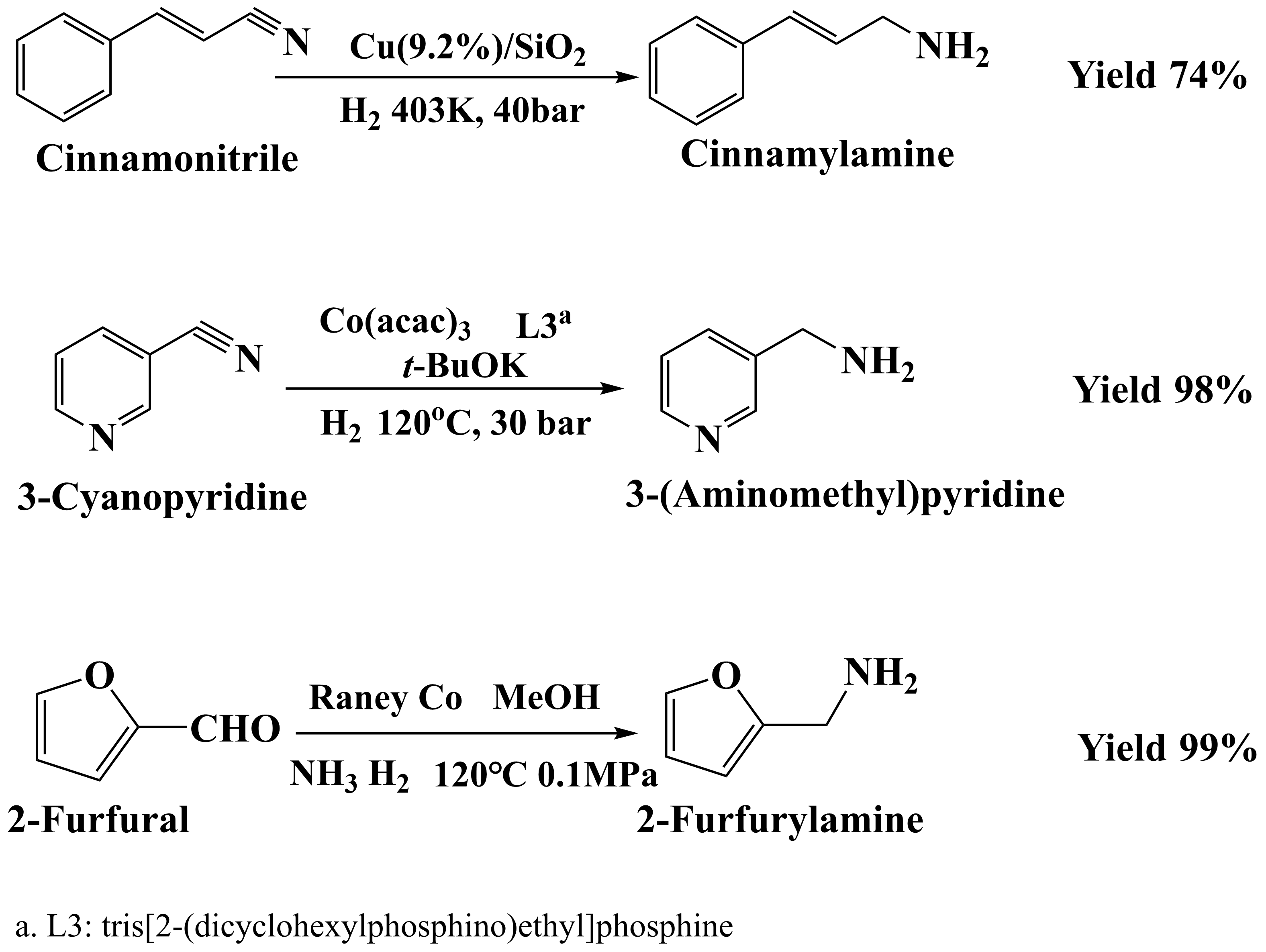

Supplement: Supplementary file 1 — Additional file 1: Fig. S1. Chemical synthesis routes of several APAs. Fig. S2. Volcano plot of DEGs between S003 and S009. Fig. S3. DEGs in TCA cycle pathway between S003 and S009. Fig. S4. DEGs in oxidative phosphorylation pathway between S003 and S009. Fig. S5. DEGs in pyruvate metabolism pathway between S003 and S009. Fig. S6. Volcano plot of DEGs between S003 and S010. Fig. S7. The effect of DMSO on the conversion of cinnamic acid to cinnamylamine by ncCAR and OATA. Fig. S8. The effect of L-Ala on the conversion of cinnamic acid to cinnamylamine by ncCAR and OATA. Table S1. Sequences of P1,6 and P2,51 promoter. Table S2. The effect of DMSO on yield, conversion and selectivity. Table S3. The effect of L-Ala on yield, conversion and selectivity. [file 13036_2023_334_MOESM1_ESM.zip › Fig.S1.tif]

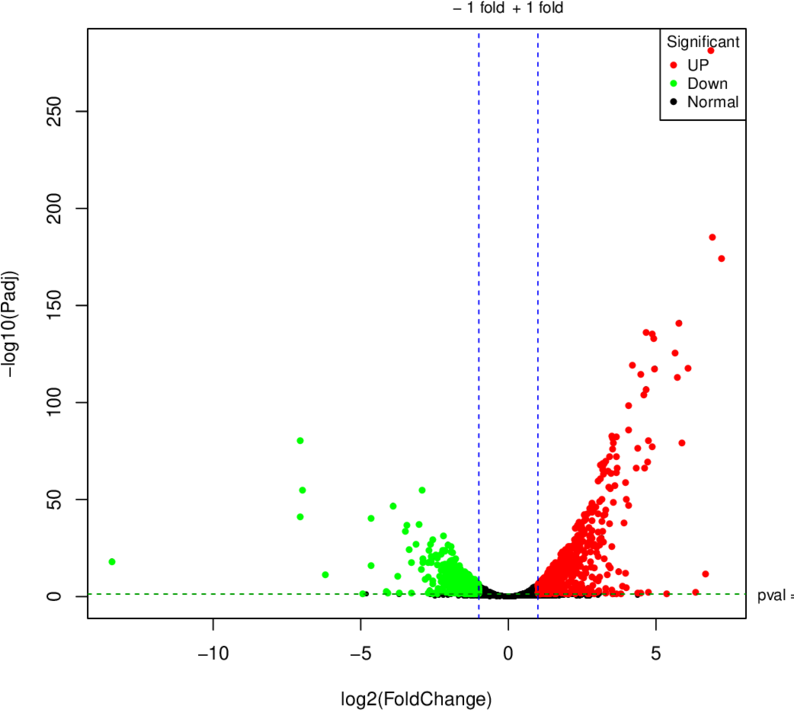

Supplement: Supplementary file 1 — Additional file 1: Fig. S1. Chemical synthesis routes of several APAs. Fig. S2. Volcano plot of DEGs between S003 and S009. Fig. S3. DEGs in TCA cycle pathway between S003 and S009. Fig. S4. DEGs in oxidative phosphorylation pathway between S003 and S009. Fig. S5. DEGs in pyruvate metabolism pathway between S003 and S009. Fig. S6. Volcano plot of DEGs between S003 and S010. Fig. S7. The effect of DMSO on the conversion of cinnamic acid to cinnamylamine by ncCAR and OATA. Fig. S8. The effect of L-Ala on the conversion of cinnamic acid to cinnamylamine by ncCAR and OATA. Table S1. Sequences of P1,6 and P2,51 promoter. Table S2. The effect of DMSO on yield, conversion and selectivity. Table S3. The effect of L-Ala on yield, conversion and selectivity. [file 13036_2023_334_MOESM1_ESM.zip › Fig.S2.tiff]

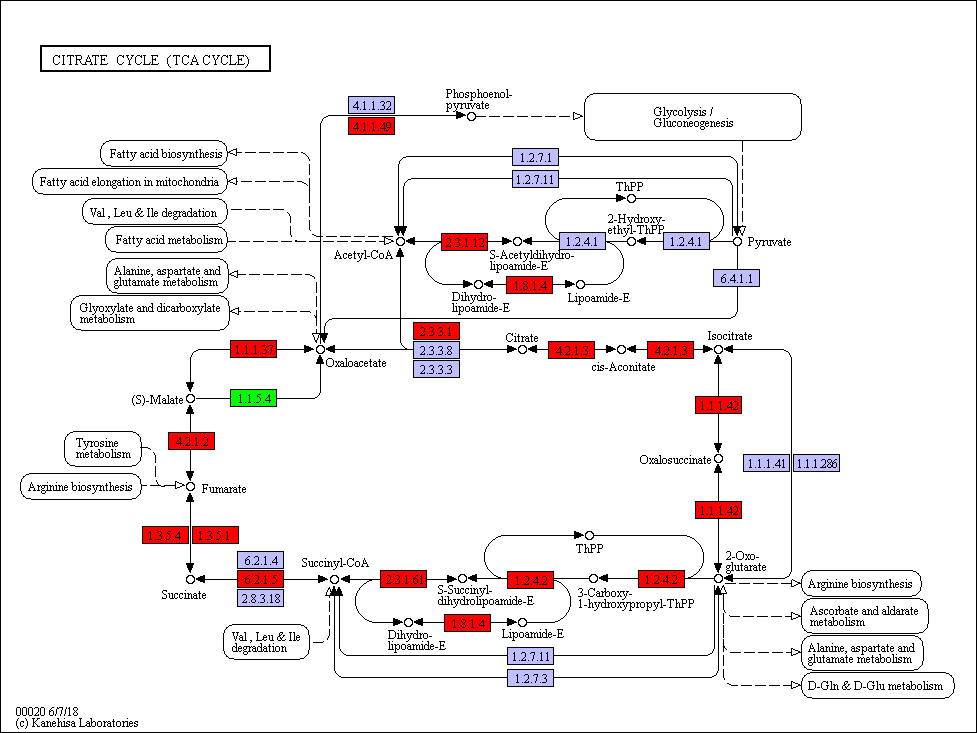

Supplement: Supplementary file 1 — Additional file 1: Fig. S1. Chemical synthesis routes of several APAs. Fig. S2. Volcano plot of DEGs between S003 and S009. Fig. S3. DEGs in TCA cycle pathway between S003 and S009. Fig. S4. DEGs in oxidative phosphorylation pathway between S003 and S009. Fig. S5. DEGs in pyruvate metabolism pathway between S003 and S009. Fig. S6. Volcano plot of DEGs between S003 and S010. Fig. S7. The effect of DMSO on the conversion of cinnamic acid to cinnamylamine by ncCAR and OATA. Fig. S8. The effect of L-Ala on the conversion of cinnamic acid to cinnamylamine by ncCAR and OATA. Table S1. Sequences of P1,6 and P2,51 promoter. Table S2. The effect of DMSO on yield, conversion and selectivity. Table S3. The effect of L-Ala on yield, conversion and selectivity. [file 13036_2023_334_MOESM1_ESM.zip › Fig.S3.tiff]

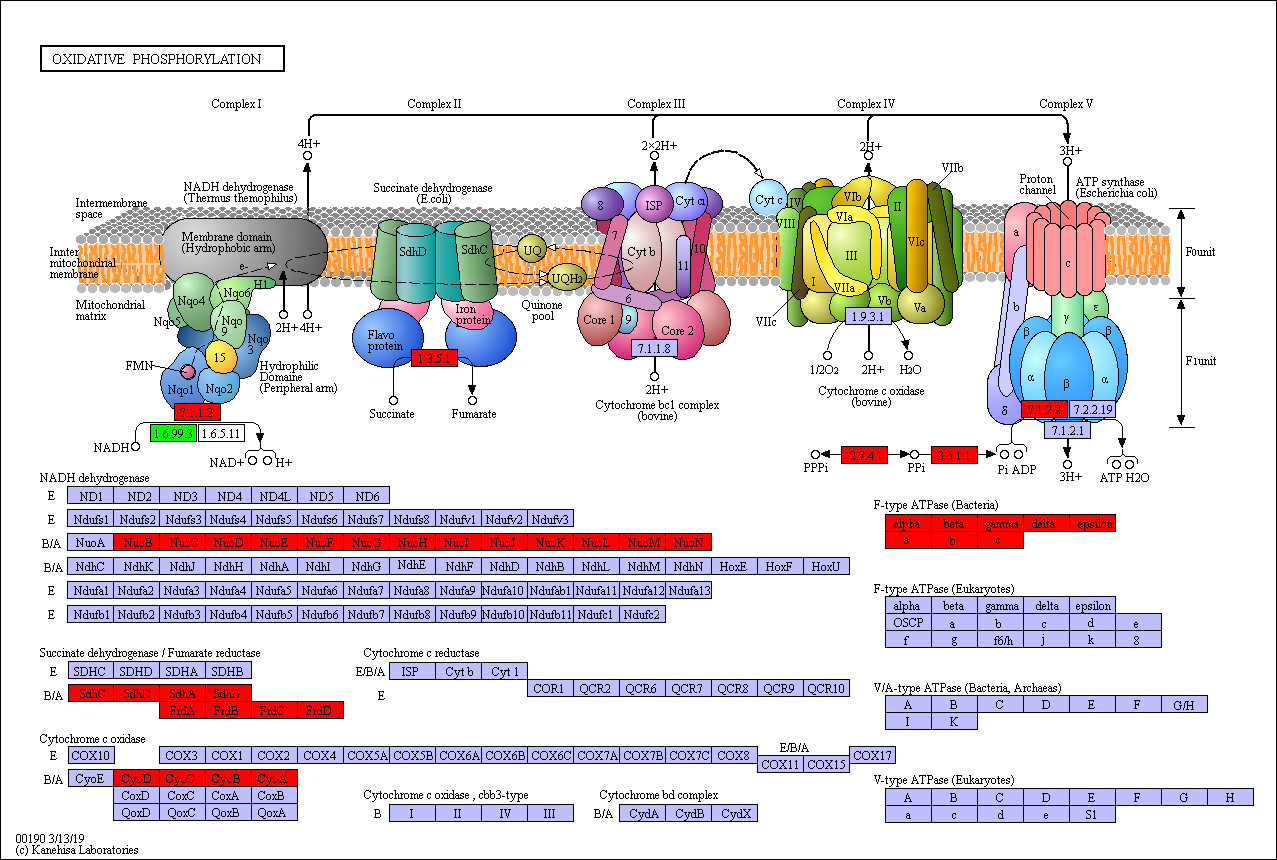

Supplement: Supplementary file 1 — Additional file 1: Fig. S1. Chemical synthesis routes of several APAs. Fig. S2. Volcano plot of DEGs between S003 and S009. Fig. S3. DEGs in TCA cycle pathway between S003 and S009. Fig. S4. DEGs in oxidative phosphorylation pathway between S003 and S009. Fig. S5. DEGs in pyruvate metabolism pathway between S003 and S009. Fig. S6. Volcano plot of DEGs between S003 and S010. Fig. S7. The effect of DMSO on the conversion of cinnamic acid to cinnamylamine by ncCAR and OATA. Fig. S8. The effect of L-Ala on the conversion of cinnamic acid to cinnamylamine by ncCAR and OATA. Table S1. Sequences of P1,6 and P2,51 promoter. Table S2. The effect of DMSO on yield, conversion and selectivity. Table S3. The effect of L-Ala on yield, conversion and selectivity. [file 13036_2023_334_MOESM1_ESM.zip › Fig.S4.tiff]

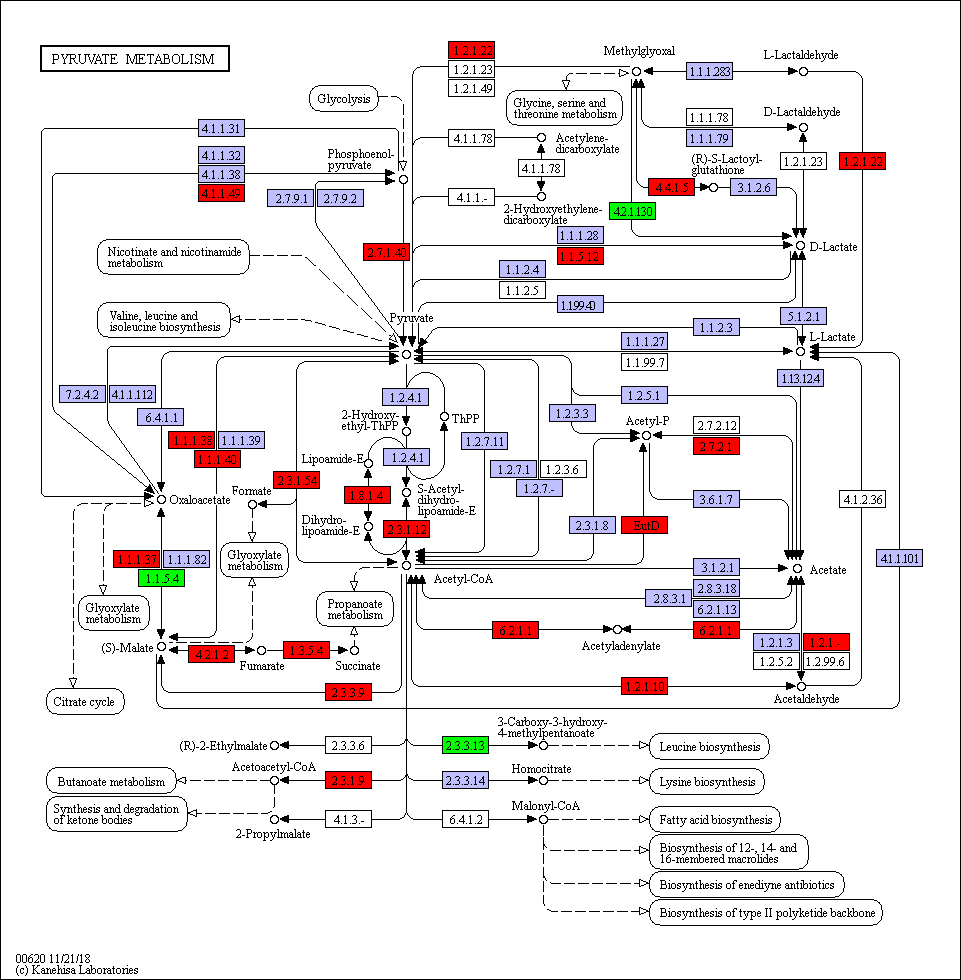

Supplement: Supplementary file 1 — Additional file 1: Fig. S1. Chemical synthesis routes of several APAs. Fig. S2. Volcano plot of DEGs between S003 and S009. Fig. S3. DEGs in TCA cycle pathway between S003 and S009. Fig. S4. DEGs in oxidative phosphorylation pathway between S003 and S009. Fig. S5. DEGs in pyruvate metabolism pathway between S003 and S009. Fig. S6. Volcano plot of DEGs between S003 and S010. Fig. S7. The effect of DMSO on the conversion of cinnamic acid to cinnamylamine by ncCAR and OATA. Fig. S8. The effect of L-Ala on the conversion of cinnamic acid to cinnamylamine by ncCAR and OATA. Table S1. Sequences of P1,6 and P2,51 promoter. Table S2. The effect of DMSO on yield, conversion and selectivity. Table S3. The effect of L-Ala on yield, conversion and selectivity. [file 13036_2023_334_MOESM1_ESM.zip › Fig.S5.tiff]

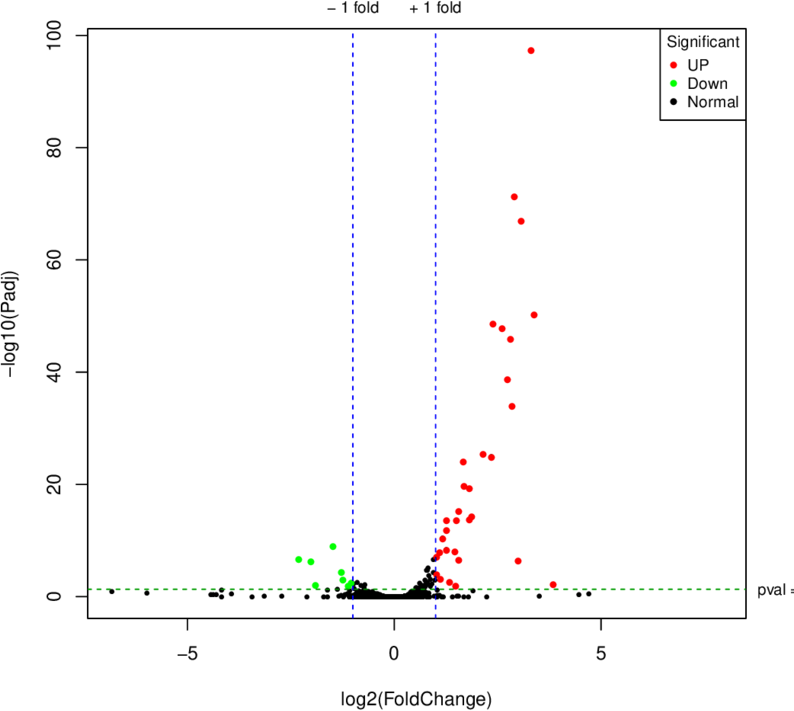

Supplement: Supplementary file 1 — Additional file 1: Fig. S1. Chemical synthesis routes of several APAs. Fig. S2. Volcano plot of DEGs between S003 and S009. Fig. S3. DEGs in TCA cycle pathway between S003 and S009. Fig. S4. DEGs in oxidative phosphorylation pathway between S003 and S009. Fig. S5. DEGs in pyruvate metabolism pathway between S003 and S009. Fig. S6. Volcano plot of DEGs between S003 and S010. Fig. S7. The effect of DMSO on the conversion of cinnamic acid to cinnamylamine by ncCAR and OATA. Fig. S8. The effect of L-Ala on the conversion of cinnamic acid to cinnamylamine by ncCAR and OATA. Table S1. Sequences of P1,6 and P2,51 promoter. Table S2. The effect of DMSO on yield, conversion and selectivity. Table S3. The effect of L-Ala on yield, conversion and selectivity. [file 13036_2023_334_MOESM1_ESM.zip › Fig.S6.tiff]
